# ﻿Phylogeny of the *Harnischia* generic complex (Diptera, Chironomidae) inferred from twenty whole mitogenomes

**DOI:** 10.3897/zookeys.1266.162901

**Published:** 2026-01-19

**Authors:** Xinyu Ge, Jiaxin Nie, Yaning Tang, Ziming Shao, Wenbin Liu, Chuncai Yan

**Affiliations:** 1 Tianjin Key Laboratory of Conservation and Utilization of Animal Diversity, Tianjin Normal University, Tianjin, 300387, China Tianjin Normal University Tianjin China

**Keywords:** Chironominae, comparative mitogenome, phylogenetic analysis, systematics, taxonomy

## Abstract

This study presents the first comprehensive mitochondrial genome analysis of three Chironomidae genera, including sequencing, assembling, and annotating mitogenomes from two *Paracladopelma* species, one *Parachironomus* species and one *Harnischia* species. These newly characterized mitogenomes were subjected to comparative genomic analysis alongside twenty previously published Chironomidae mitogenomes, enabling robust phylogenetic reconstruction within the *Harnischia* generic complex. The Ka/Ks ratio analysis reveals that most protein-coding genes (PCGs) have undergone purifying selection, with *ND6*, *ATP8*, and *ND5* exhibiting higher ω values and thus greater evolutionary flexibility. Phylogenetic analyses using Bayesian inference and Maximum likelihood methods demonstrate *Robackia* as a basal group. This study expands the available mitogenomic database and provides a robust foundation for future phylogenetic analyses of the *Harnischia* generic complex.

## ﻿Introduction

Chironomidae, comprising approximately 15,000 species, are adept at colonizing diverse aquatic habitats, from low-oxygen waters to Himalayan ice and Lake Baikal’s great depth (Armitage et al. 1995). Their resilience in extreme conditions, coupled with their wide distribution, renders them valuable bioindicators for ecological and environmental assessments (Ferrington 2008). With a significant role in detritus processing and trophic dynamics, high population density, and unique life cycle traits, they are valuable for both ecological research and biological monitoring (Pinder 1986; Cranston et al. 1989; Armitage et al. 2012). In taxonomy, the family Chironomidae is divided into 11 subfamilies, among which Chironominae is the largest (Andersen et al. 2013).

The *Harnischia* generic complex, of the subfamily Chironominae comprises over 320 species in 20 genera worldwide, and is marked by wide distribution, high population density, and significant biomass (Orel et al. 2017; Mukherjee and Hazra 2023). Since the concept of the generic complex was introduced in 1969 (Beck and Beck 1969), significant taxonomic progress has been made, notably through Townes’ revision of male adults of Chironomini in North America, which differentiated the complex into the genera *Harnischia* and *Cryptochironomus* Kieffer, 1918 (Townes 1945). Later, Sæther’s global-scale research established nine genera and explored their phylogenetic relationships morphologically, supporting the *Harnischia* complex’s monophyly within the family Chironomidae (Sæther 1977a, b). However, phylogenetic research on the *Harnischia* complex remains limited, with ongoing debates about its generic boundaries and taxonomic status, including issues related to monotypic genera. Despite their morphological similarity, *Paracladopelma* Harnisch, 1923 and *Parachironomus* Lenz, 1921 exhibit less close genetic relationships based on short genetic fragment studies, and their phylogenetic relationship has long been controversial (Yan 2007; Cranston et al. 2011; Andersen et al. 2017).

The mitochondrial genomes (mitogenomes) of insects have recently gained significant attention in research due to their highly conserved structure, which closely resembles that of ancestral insects (Cameron 2014). These mitogenomes are typically double-stranded circular molecules, varying in size from 14 to 20 kb. They encode a full set of genetic components, including 13 protein-coding genes (PCGs), two ribosomal RNAs (rRNAs), 22 transfer RNAs (tRNAs), and a control region (CR) (Brown 1985; Boore 1999; Ge et al. 2022; Ge et al. 2024). Given their small size, maternal inheritance, low recombination rates, and rapid evolution, insect mitogenomes serve as powerful tools for molecular identification and phylogenetic analysis across Diptera, particularly within the family Chironomidae and even the *Harnischia* generic complex (Song et al. 2019; Li et al. 2022; Lin et al. 2022; Liu et al. 2024; Liu et al. 2025).

In this study, we have sequenced, assembled, and annotated the mitogenomes of two *Paracladopelma* species, one *Parachironomus* species, and one *Harnischia* Kieffer, 1921 species. The mitogenome of *Paracladopelma* is first reported publicly. Additionally, we incorporated twenty previously published mitogenomes into our analysis to delve deeper into their characteristics. Utilizing Bayesian inference (BI) and Maximum likelihood (ML) methods across various databases, we reconstructed the phylogenetic relationships among genera within the *Harnischia* generic complex. Our findings indicate the sister-group relationship between *Paracladopelma* and *Parachironomus*. In addition, *Robackia* is a basal group of *Harnischia* generic complex, while *Cryptochironomus* + *Demicryptochironomus* Lenz, 1941 are terminal groups.

## ﻿Materials and methods

### ﻿Sampling and sequencing

The sample collection is shown in Table 1. Species were identified using an integrative approach combining morphological assessment and DNA barcoding. The morphological characteristics of the four species under scrutiny conform to the descriptions provided in references (Yan et al. 2012; Mukherjee and Hazra 2023; Liu et al. 2023). Prior to DNA extraction and morphological analysis, all specimens were preserved in 85–95% ethanol at a temperature of −20 °C. Genomic DNA was systematically isolated from thoracic and leg tissues utilizing the TIANamp Genomic DNA Kit. The voucher specimens have been accessioned into the permanent collection of the College of Life Sciences at TJNU, Tianjin, China.

**Table 1.** Collection details for the newly sequenced species.

Species	Sample ID	Location	Latitude and Longitude	Date	Collector
*Harnischia inawabeceus*	KYX737	Yunhe, Zhejiang, China	28°7'6.7"N, 119°34'6.2"E	29 July 2012	Liu Wenbin
*Parachironomus* sp.	XBZ165	Xishuangbanna Tropical Botanical Garden, Yunnan, China	22°0'38.7"N, 100°47'45.6"E	25 April 2014	Wang Qiang
*Paracladopelma camptolabis*	KLF008	Furong Town, Zhejiang, China	28°6'55.6"N, 120°59'0.2"E	17 July 2011	Lin Meixin
*Paracladopelma* sp.	3QL001	Haibei Tibetan Autonomous Prefecture, Qinghai, China	38°10'37.1"N, 100°15'5.9"E	29 July 2020	Ge Xinyu, Peng Lang

To amplify the 658 bp segment of the *mtCOI* barcode region (Hebert et al. 2003), we employed the universal primers LCO1490 and HCO2198 (Ge et al. 2022; Ge et al. 2024). Subsequently, it was sent to BGI TECH SOLUTIONS (BEIJING LIUHE) CO., LIMITED for Sanger sequencing. Library preparation was performed with a 350 bp insert size, followed by paired-end 150 bp reads using the Illumina NovaSeq XPlus platform.

### ﻿Assembly, annotation and composition analyses

Mitogenomes assembly was performed de novo using NOVOPlasty v. 3.8.3 (Dierckxsens et al. 2017), with the *mtCOI* barcode sequence serving as the initial seed. To optimize assembly parameters, we used k-mer sizes of 39 bp (Ge et al. 2022). The secondary structures of tRNAs were predicted and analyzed using the MITOS2 WebServer (Donath et al. 2019), enabling detailed characterization of their structural conformations. The PCGs were annotated using Geneious Prime v. 2025.0.1 by identifying open reading frames (ORFs) with reference to the invertebrate mitogenomic code. The boundaries between PCGs and rRNAs were subsequently manually verified through alignment with reference sequences. Moreover, the synonymous (Ks) and non-synonymous substitution rates (Ka) of PCGs were computed using DnaSP v. 6.0 (Rozas et al. 2017). MEGA X was employed to determine the relative synonymous codon usage of the mitogenomes, and nucleotide composition bias was analyzed using SeqKit v. 0.16.0 (Shen et al. 2016). The mitogenomes map was generated via the CGView server (accessible at https://cgview.ca/, accessed on 8 May 2025).

### ﻿Phylogenetic analyses

A total of 13 PCGs and 2 rRNAs of 20 mitogenomes were retrieved from GenBank for phylogenetic analyses. This comprehensive dataset encompassed 24 subfamily Chironominae species, and two *Chironomus* and two *Polypedilum* species were used as outgroups (Andersen et al. 2017; Liu et al. 2021). Sequence alignment for both nucleotides and proteins was performed using MAFFT v. 7.470 with the L-INS-I algorithm (Katoh and Standley 2013). Subsequently, sequence trimming was carried out with Trimal v. 1.4.1 (-automated1) (Capella-Gutiérrez et al. 2009), in preparation for phylogenetic analysis.

The phylogenetic analysis was based on five matrices generated by FASconCAT-G v. 1.05 (Kück and Longo 2014). These matrices were configured as follows: (1) cds_fna, which encompassed all codon positions of the 13 PCGs; (2) cds_rrna, integrating all codon positions of the 13 PCGs along with the rRNA sequences; (3) cds12_rrna, incorporating the first and second codon positions of the 13 PCGs and the two rRNA sequences; (4) cds12_fna, focusing solely on the first and second codon positions of the 13 PCGs; and (5) cds_faa, utilizing the amino acid sequences derived from the 13 PCGs. Matrix heterogeneity was assessed using AliGROOVE v. 1.0.6 with analytical parameters standardized according to previous methodologies (Kück et al. 2014).

The phylogenetic reconstruction was conducted using both Bayesian inference (BI) and maximum likelihood (ML) methods. For the ML analysis, the optimal substitution model was selected using MODELFINDER (Kalyaanamoorthy et al. 2017) implemented in IQ-TREE v. 2.2.0.8 (Minh et al. 2020) with 1000 replicates of UFBoot2 (-alrt 1000) and 1000 replicates of SH-aLRT (-B 1000) (Hoang et al. 2018). For the BI analysis, the CAT+GTR site-heterogeneous mixture model in Phylobayes-MPI v. 1.9 (Lartillot et al. 2013) was employed. After discarding the first 25% of generations as burn-in from two independent Markov chains, a consensus tree was constructed. The final phylogenetic trees were visualized using FigTree v. 1.4.3 (Rambaut 2018).

## ﻿Results

### ﻿Mitogenomic organization

**Figure 1.**
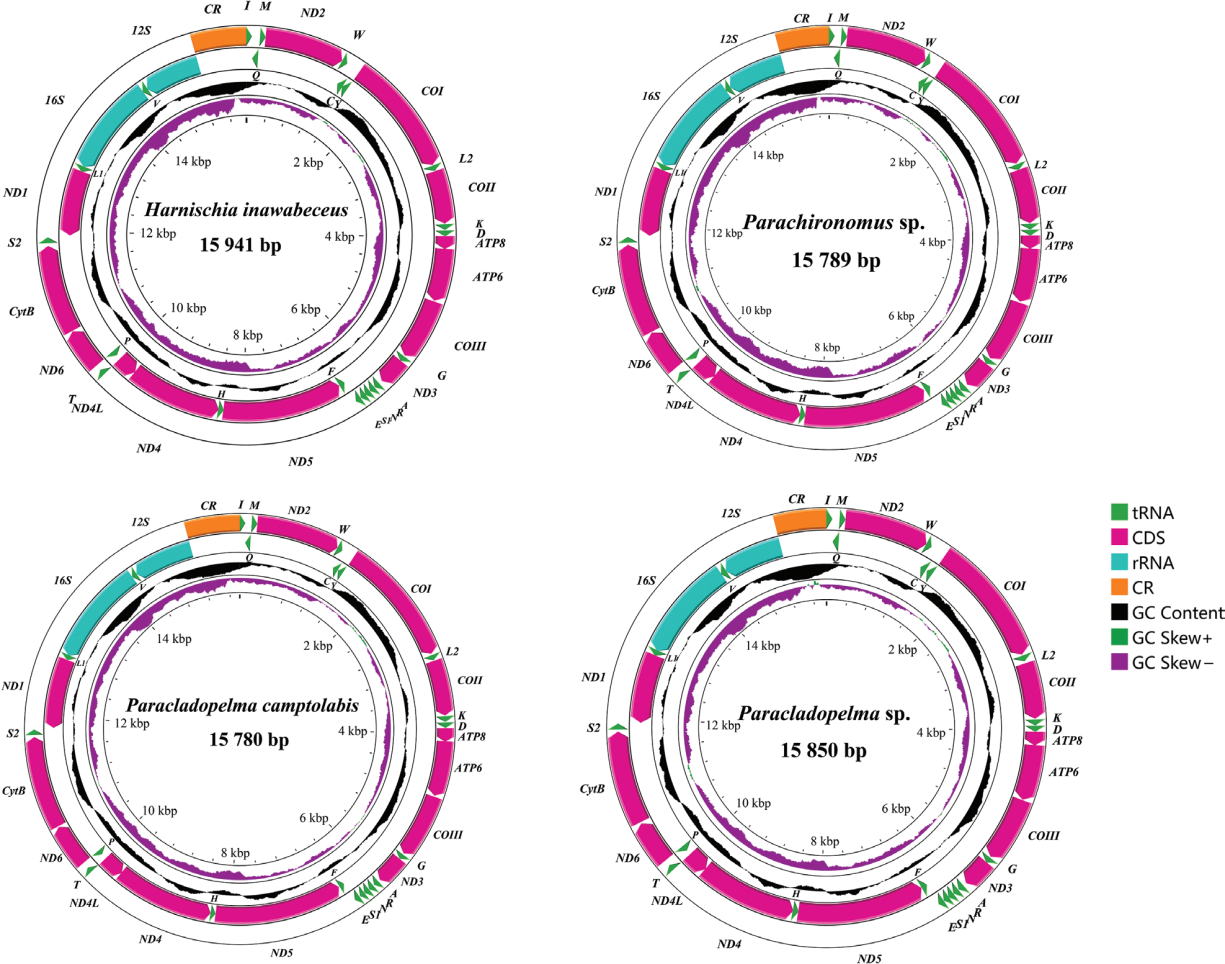
The mitogenomes map clearly illustrates the characteristics of the mitogenomes of representative species from four genera within the *Harnischia* complex. The map uses arrows to denote gene transcription direction and employs standard abbreviations for PCGs and rRNAs, along with simplified tRNA notations, for clarity. The second circle displays GC content, providing insights into its nucleotide composition, while the third circle shows GC-skew, highlighting structural asymmetry. The innermost layer indicates the mitogenome’s length, providing a comprehensive perspective for understanding its genomic characteristics.

Each complete mitogenome contains 37 genes—13 PCGs, 22 tRNAs, two rRNAs and a control region (CR). The complete mitogenome of *Harnischia inawabeceus* Sasa, Kitami & Suzuki, 1999 was 15,941 bp, *Parachironomus* sp. was 15,789 bp, *Paracladopelma camptolabis* Kieffer, 1913 was 15,780 bp and *Paracladopelma* sp. was 15,850 bp long (Fig. 1). The lengths of most of these newly assembled mitogenomes were comparable to those of previously published Chironomidae mitogenomes.

### ﻿Protein-coding genes, codon usage, and evolutionary rates

**Figure 2.**
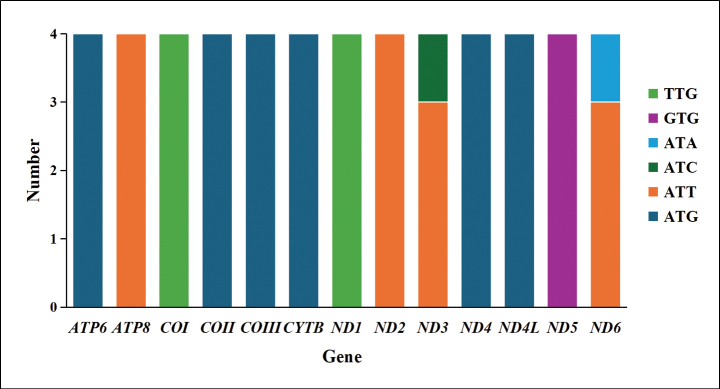
Start codons of PCGs among *Harnischia* generic complex mitogenomes.

All 13 PCGs in the newly assembled mitogenomes predominantly use the canonical start codon ATN, aligning closely with the typical mitochondrial start codon observed in insects. However, deviations were noted in other genes. Specifically, the *COI* and *ND1* genes used TTG as the start codon in four species. The *ATP8* gene began with ATT in four species. Similarly, the *ND2* gene started with ATT in all species, and the *ND3* gene began with ATT in three species and ATC in one species. Furthermore, the *COII*, *COIII*, *CYTB*, *ND4*, *ATP6* gene and *ND4L* genes consistently started with ATG. The *ND5* gene uniquely started with GTG in four species, while the *ND6* gene exclusively began with ATT in three species and ATA in one species (Fig. 2).

**Figure 3.**
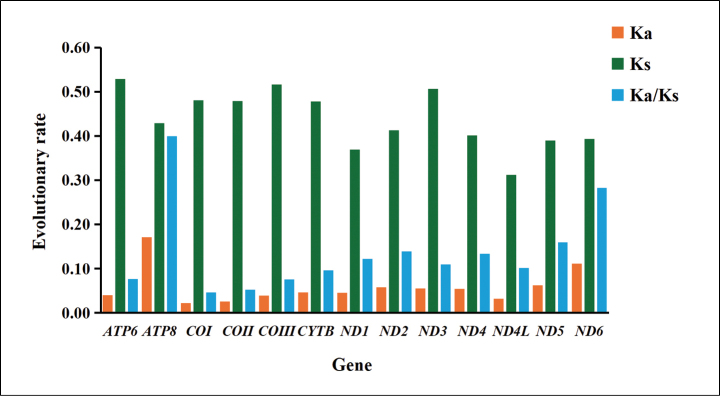
Evolutionary rates of the 13 PCGs in the *Harnischia* generic complex. Non-synonymous substitutions are denoted as Ka, while synonymous substitutions are represented as Ks. The Ka/Ks ratio indicates the selection pressure on each PCG. The *x*-axis lists the 13 PCGs, and the *y*-axis displays the Ka/Ks values.

The Ka/Ks ratio, also known as ω, is a widely used metric for quantifying the rate of sequence evolution in the context of natural selection. Our findings closely mirror those reported for other insect species, demonstrating that the Ka/Ks values for all PCGs consistently fell below one, with a range spanning from 0.046 for *COI* to 0.400 for the *ATP8* (Fig. 3). The evolutionary rates of these PCGs can be ordered as follows: *ATP8* > *ND6* > *ND5* > *ND2* > *ND4* > *ND1* > *ND3* > *ND4L* > *CYTB* > *ATP6* > *COIII* > *COII* > *COI*. Our findings reveal that a substantial proportion of these genes have undergone purifying selection, a process that eliminates deleterious mutations and is modulated by differential selective pressures. The low ω ratios observed in *COII* and *COI* reflect stringent selective constraints, indicative of strong evolutionary conservation. In contrast, *ND6*, *ATP8* and *ND5* exhibit higher ω values, suggesting a more permissive selective regime and greater evolutionary flexibility (Fig. 3). These results provide valuable insights into the evolutionary trajectories of PCGs and underscore the influence of natural selection on their sequence divergence.

### ﻿Phylogenetic analysis

#### ﻿Heterogeneity analysis

The heterogeneity analysis revealed distinct patterns of sequence similarity in mitogenomes across different species. Due to the degeneracy of the genetic code, the cds_faa exhibited the lowest level of heterogeneity, whereas the cds12_rrna and cds_rrna demonstrated relatively higher degrees of heterogeneity.

**Figure 4.**
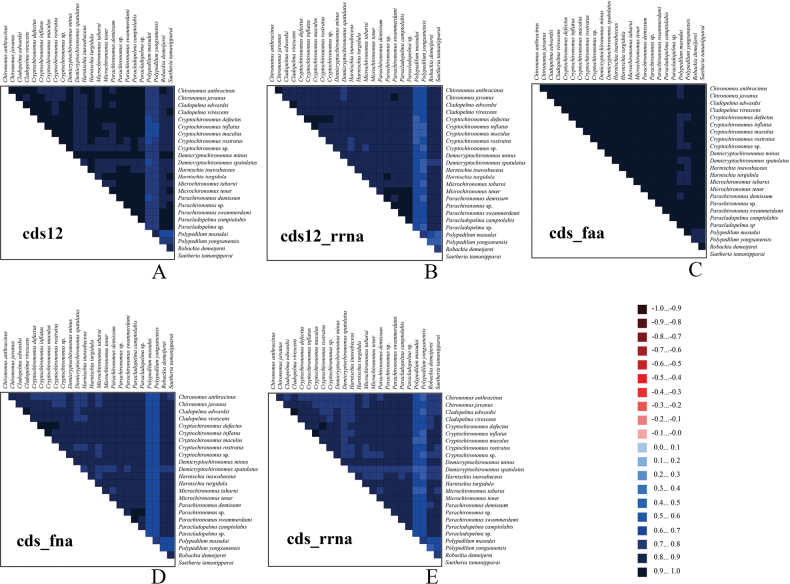
The evaluation of genomic variation across the mitogenomes of 24 species within the *Harnischia* complex primarily examined their PCGs, amino acid sequences, and rRNAs. Sequence conservation patterns were visualized using a color-coded system based on AliGROOVE indices, where values range from −1 (denoting pronounced sequence divergence, marked in red) to +1 (indicating high sequence conservation, shown in blue). In this representation, lighter-colored blocks reflect greater sequence dissimilarity, whereas darker shades correspond to higher levels of sequence uniformity. **A.** cds12_fna; **B.** cds12_rrna; **C.** cds_faa; **D.** cds_fna; **E.** cds_rrna.

Within PCGs, the mutation rate at the third codon position was significantly higher than that at the first and second positions. Consequently, the third codon position was deemed unsuitable for inferring phylogenetic relationships within the *Harnischia* generic complex (Fig. 4).

This study integrates the strengths of Bayesian inference (BI) and maximum likelihood (ML) methods, constructing ten phylogenetic trees using five distinct datasets (figs S1–S9). Mitogenome data support the inclusion of the newly sequenced species of *Harnischia inawabeceus*, *Parachironomus* sp., *Paracladopelma camptolabis*, and *Paracladopelma* sp. in the *Harnischia* generic complex (Fig. 5).

**Figure 5.**
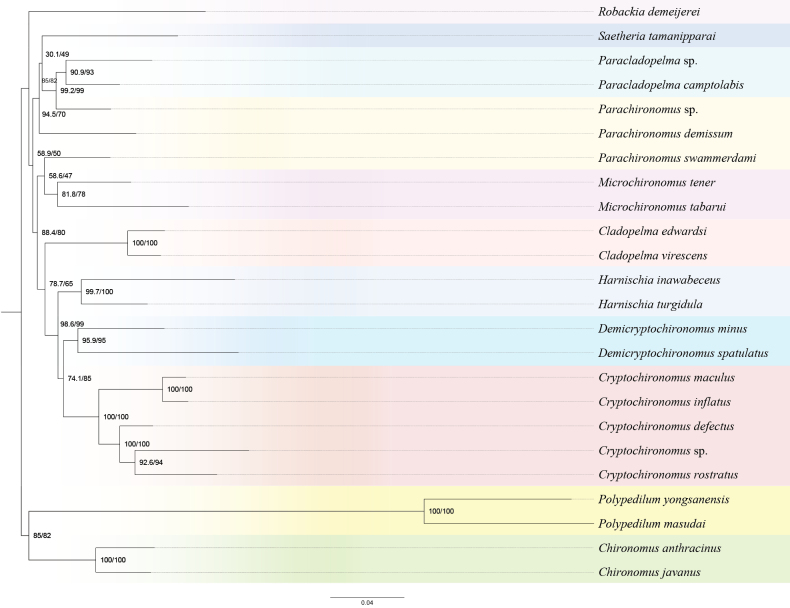
Phylogenetic tree of *Harnischia* generic complex, ML tree based on analysis cds_rrna in Partition.

### ﻿Systematics and phylogeny

Regarding phylogenetic analyses within the *Harnischia* complex and its relationship to other genera in the subfamily Chironominae, studies have been conducted based on mitogenomes (Liu et al. 2024; Liu et al. 2025). The addition of mitogenome data revealed that the internal phylogenetic relationships within the *Harnischia* complex reconstructed from this data remain consistent with both the congruence and discrepancies observed in previous studies utilizing characters from female adults and fragments of *18S rRNA*, *28S rRNA*, *CAD1*, *CAD4*, and *mtCOI* (Sæther 1977a; Cranston et al. 2011). Furthermore, the topological relationships among the five genera *Cryptochironomus*, *Demicryptochironomus*, *Harnischia*, *Cladopelma*, and *Microchironomus* align with earlier research findings (Kong et al. 2021; Liu et al. 2025). Additionally, *Robackia* is identified as occupying the basal position within the complex, indicating its relatively early diverging status.

In the recent phylogenetic study of Chironomidae, particularly with respect to the subfamily Chironominae, a tree based on 119 morphological characters from larval, adult and pupal stages strongly supports the monophyly of the *Harnischia* generic complex. However, this analysis places *Parachironomus*, *Demicryptochironomus* and *Paracladopelma* as basal lineages, whereas the topology inferred from mitochondrial genomes identifies *Robackia* as the most basal taxon and positions *Cryptochironomus* and *Demicryptochironomus* as derived clades (Andersen et al. 2017). In addition, a separate phylogenetic study of marine Chironomini based on only a few taxa used six genetic markers (*18S rRNA*, *28S rRNA*, *CAD1*, *CAD4*, *FolCOI* and *COI*) and fossil calibrations to produce Bayesian time-calibrated phylogenies that recover *Microchironomus* and *Paracladopelma* as sister taxa, a relationship that is robustly corroborated in multi-species mitogenomic analyses (Tang et al. 2023).

This study reports the first mitogenomic data for the genus *Paracladopelma*. Morphologically, the male hypopygia of *Paracladopelma* are closely similar to those of *Parachironomus* and *Saetheriella*, leading to ambiguous species assignment in some cases (Yan 2007). Previous phylogenetic analyses based on fragments of standard markers (e.g., *18S rRNA*, *28S rRNA*, *CAD*, *COI*) and all life stages characteristics did not recover *Paracladopelma* and *Parachironomus* as sister groups (Cranston et al. 2011; Andersen et al. 2017). Phylogenetic reconstructions recovered the three species of *Parachironomus* terminal taxa in non-contiguous positions, contradicting the morphology-based delimitation. To test the observed incongruence, we assembled all publicly available *COI* sequences for *Parachironomus* and the putatively related genus *Paracladopelma* from GenBank. Neighbour-joining analysis (Suppl. material 1: fig. S10) nested the focal *Parachironomus* sp. within a well-supported *Paracladopelma* clade. Concordantly, full mitochondrial genome comparisons returned an exceptionally low inter-generic divergence, indicating a sister-group-possibly congeneric-relationship. Morphologically, however, the specimen exhibits a digitiform superior volsella that is ≥2.5× longer than wide and carries two to three strong setae, a character combination diagnostic for the genus *Parachironomus*. Consequently, mitochondrial phylogeny and morphological taxonomy are in conflict. Pending integrative revision that includes nuclear loci and type-material verification, we conservatively refer to the specimen as *Parachironomus* sp. based on current morphological standards.

*Parachironomus demissum* was originally assigned to *Paracladopelma* (Yan et al. 2012), but was transferred to genus *Parachironomus* because its superior volsella is slender and digitiform, conforming to the emended generic diagnosis rather than pediform to rectangular and microtrichiose as in genus *Paracladopelma* (Liu et al. 2023). Our neighbour-joining analysis places *P. demissum* within a strongly supported *Parachironomus* clade, and examination of freshly collected material confirms the diagnostic volsella morphology (Suppl. material 1: fig. S10). Consequently, *P. demissum* is retained in the genus *Parachironomus*, corroborating the earlier taxonomic reassignment. Although both mitochondrial and morphological evidence indicate extensive overlap between *Parachironomus* and *Paracladopelma*, a formal synonymy requires denser taxon sampling and multi-locus nuclear data. Pending such an integrative revision, we conservatively maintain *Parachironomus* sp. and *P. demissum* under *Parachironomus*, prioritising morphological identity when molecular and morphological signals conflict.

Neighbor-joining analysis recovers *Parachironomus swammerdami* as the sister taxon to *Microchironomus* (Suppl. material 1: fig. S11), a placement that renders *Parachironomus* paraphyletic unless *Microchironomus* is subsumed within it. The species retains the diagnostic digitiform superior volsella (length/width ≥ 2.5) bearing two or three strong setae, unequivocally aligning it with the morphological characters of *Parachironomus*. Consequently, the observed topology implies that either (i) *Parachironomus* is paraphyletic with respect to *Microchironomus*, or (ii) the latter genus is nested within the former. Critically, this hypothesis rests on sparse taxon sampling; comprehensive population-level sampling and multi-locus nuclear phylogenetics are required before any nomenclatural adjustment can be proposed. Nevertheless, such a taxonomic revision would require further validation with additional species data or whole-genome sequences.

## ﻿Conclusions

Our findings provide novel insights into the phylogeny of the *Harnischia* generic complex, though further studies incorporating more species and diverse datasets are necessary to elucidate a more natural phylogenetic framework for this group. These novel mitogenomes demonstrate conserved structural and nucleotide characteristics consistent with those of established Chironomidae conspecifics, significantly enriching the mitogenomic database and establishing a robust foundation for future phylogenetic analyses.

Despite significant morphological divergence across life stages (larvae, pupae, adults) among Chironomidae, phylogenetic inferences derived from morphology, short gene sequences, and mitogenomes exhibit notable discordance. Nevertheless, molecular phylogenetics increasingly underscores the enduring value of morphological analysis in chironomid systematics. While comprehensive mitogenomic analysis holds considerable promise, it demands rigorous scrutiny. A robust systematic framework integrating morphological, biogeographic, and life-history traits across developmental stages, augmented by genomic data, remains essential for elucidating intrinsic evolutionary relationships.

## ﻿Additional information

### Use of AI

No use of AI was reported.

### Author ORCIDs

Xinyu Ge https://orcid.org/0000-0002-8409-2584

Wenbin Liu https://orcid.org/0000-0001-6916-0692

Chuncai Yan https://orcid.org/0000-0003-3518-8974

### Data availability

All of the data that support the findings of this study are available in the main text or Supplementary Information.

## Supplementary materials

Supplementary material 1 Supplementary figures and tables Data type zip Explanation note **figure S1.** Phylogenetic tree of *Harnischia* generic complex, BI tree based on analysis cds_faa in Partition; **figure S2.** Phylogenetic tree of *Harnischia* generic complex, ML tree based on analysis cds_faa in Partition; **figure S3.** Phylogenetic tree of *Harnischia* generic complex, BI tree based on analysis cds_fna in Partition; **figure S4.** Phylogenetic tree of *Harnischia* generic complex, ML tree based on analysis cds_fna in Partition; **figure S5.** Phylogenetic tree of *Harnischia* generic complex, BI tree based on analysis cds_rrna in Partition; **figure S6.** Phylogenetic tree of *Harnischia* generic complex, ML tree based on analysis cds_rrna in Partition; **figure S7.** Phylogenetic tree of *Harnischia* generic complex, BI tree based on analysis cds12_fna in Partition; **figure S8.** Phylogenetic tree of *Harnischia* generic complex, ML tree based on analysis cds12_fna in Partition; **figure S9.** Phylogenetic tree of *Harnischia* generic complex, BI tree based on analysis cds12_rrna in Partition; **figure S10.** Phylogenetic trees of *Parachironomus* and *Paracladopelma* was constructed using the Neighbor-Joining method based on the *COI* gene sequences; **figure S11.** Phylogenetic trees of *Parachironomus* and *Microchironomus* was constructed using the Neighbor-Joining method based on the *COI* gene sequences; **table S1.** Final gene partitions for the Maximum Likelihood Phylogenetic analysis; **table S2.** Mitogenomes of the 24 species used in this study; **table S3.** Nucleotide composition of 4 mitogenomes. https://binary.pensoft.net/file/1514998This dataset is made available under the Open Database License (http://opendatacommons.org/licenses/odbl/1.0/). The Open Database License (ODbL) is a license agreement intended to allow users to freely share, modify, and use this Dataset while maintaining this same freedom for others, provided that the original source and author(s) are credited.Xinyu Ge, Jiaxin Nie, Yaning Tang, Ziming Shao, Wenbin Liu, Chuncai Yan
