# Supplementary material for: ﻿Phylogeny of the Harnischia generic complex (Diptera, Chironomidae) inferred from twenty whole mitogenomes
Source: Zookeys. 2026 Jan 19;1266:353–66. doi: 10.3897/zookeys.1266.162901 (PMC12835873; doi:10.3897/zookeys.1266.162901)
Supplement: Supplementary material 1 — Supplementary figures and tables [file zookeys-1266-353_article-162901__-s001.zip › Supplementary Materials/Supplementary Materials.docx]

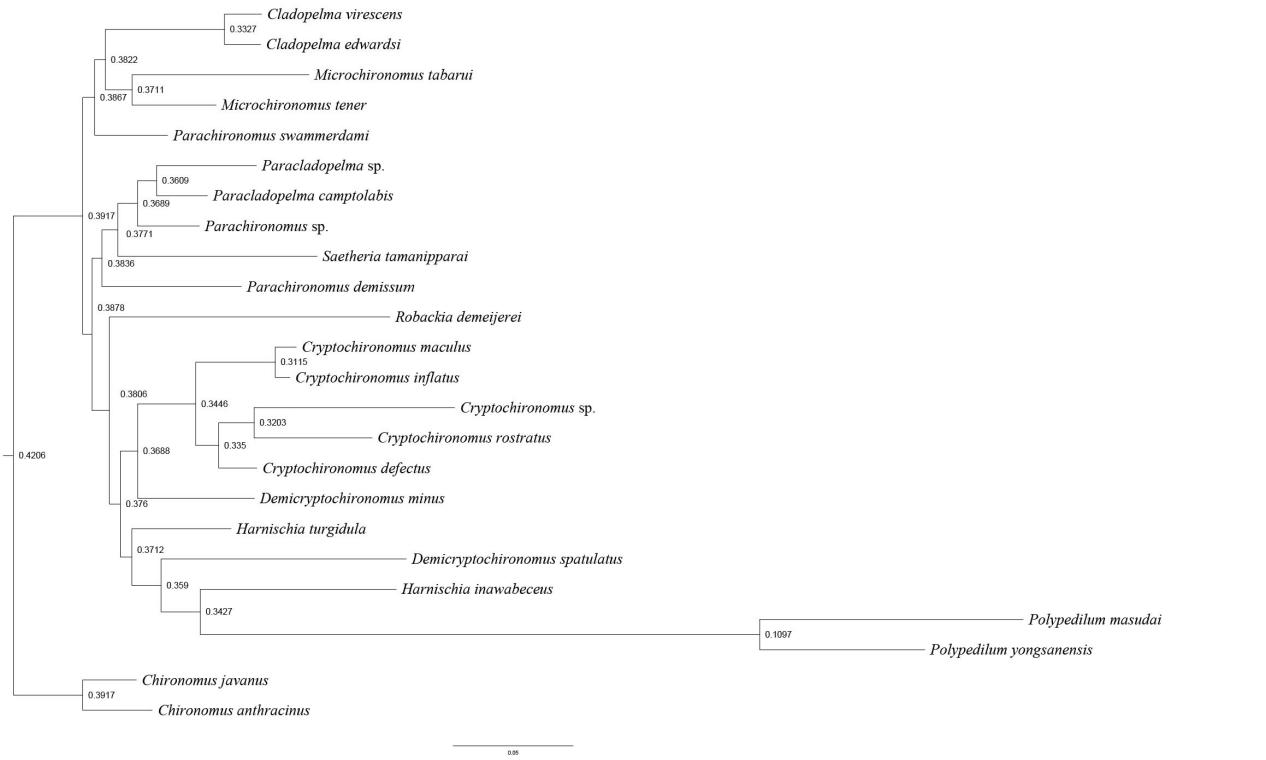


**Figure S1.** Phylogenetic tree of *Harnischia* generic complex, BI tree based on analysis cds_faa in Partition.


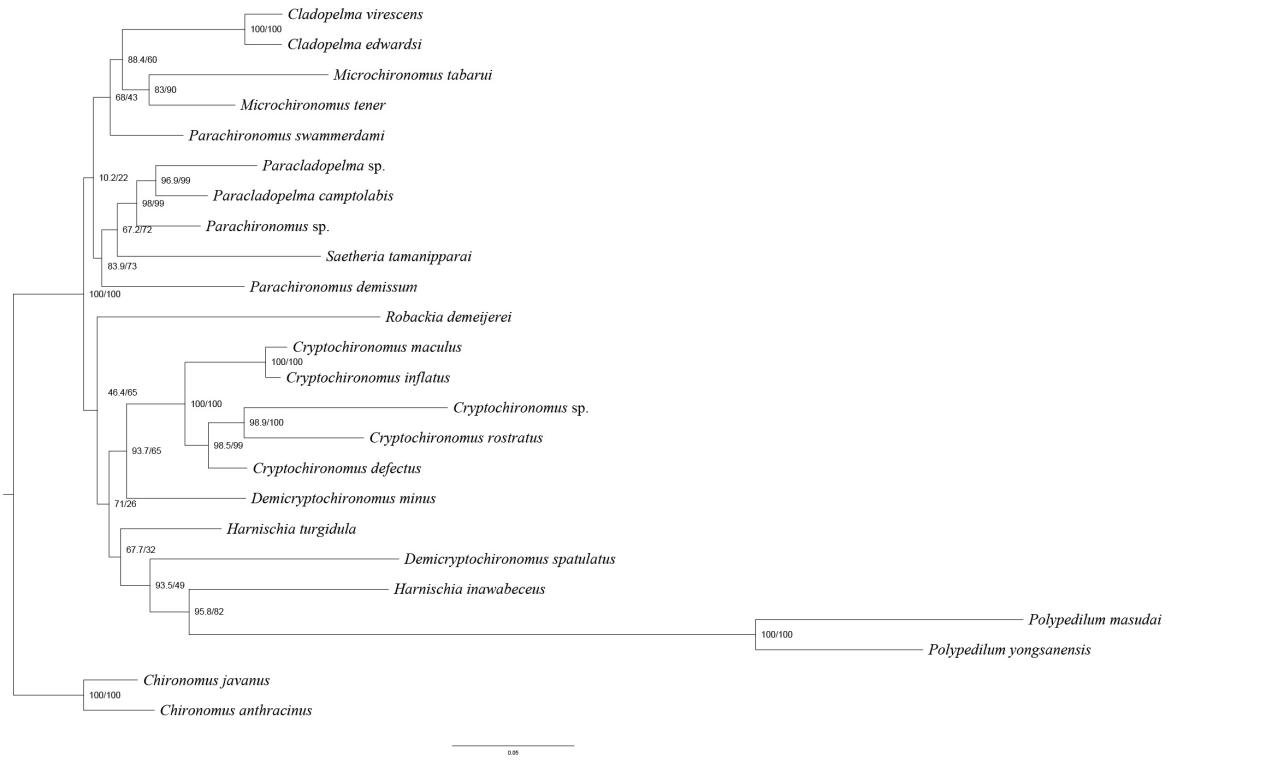


**Figure S2.** Phylogenetic tree of *Harnischia* generic complex, ML tree based on analysis cds_faa in Partition.


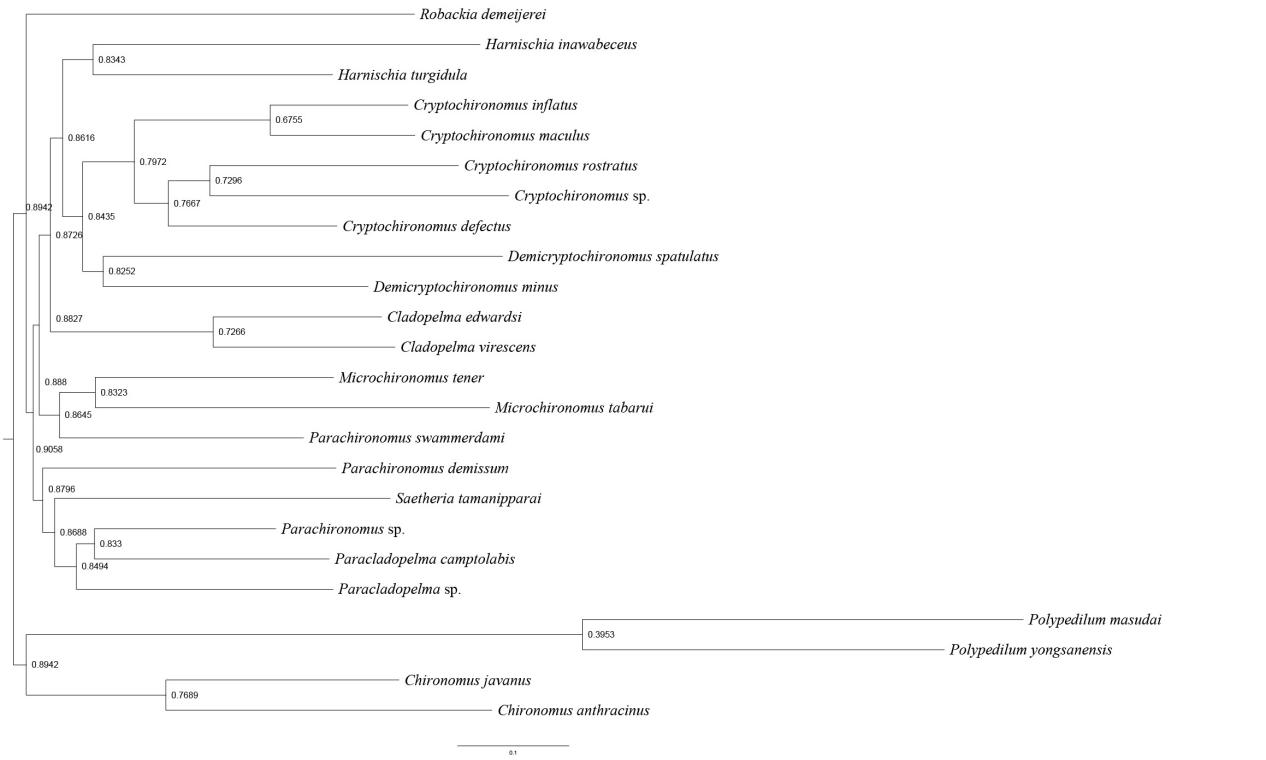


**Figure S3.** Phylogenetic tree of *Harnischia* generic complex, BI tree based on analysis cds_fna in Partition.


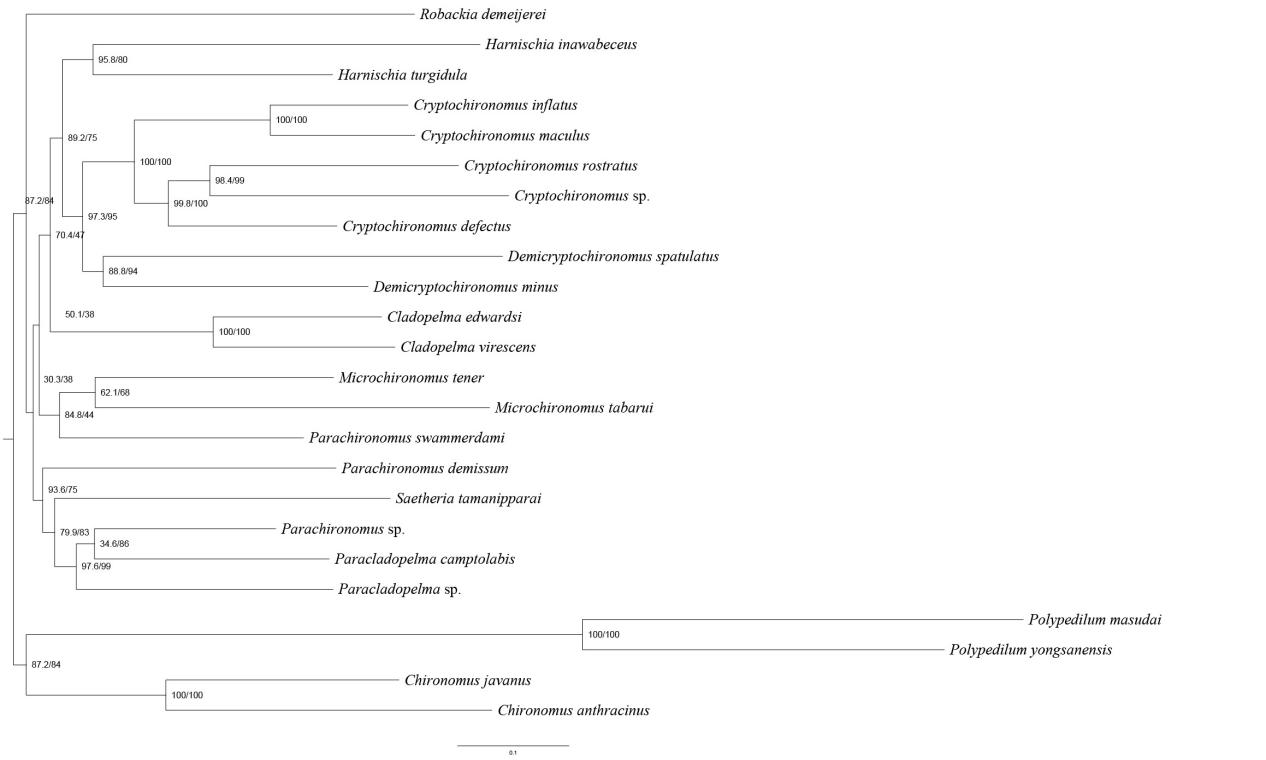


**Figure S4.** Phylogenetic tree of *Harnischia* generic complex, ML tree based on analysis cds_fna in Partition.


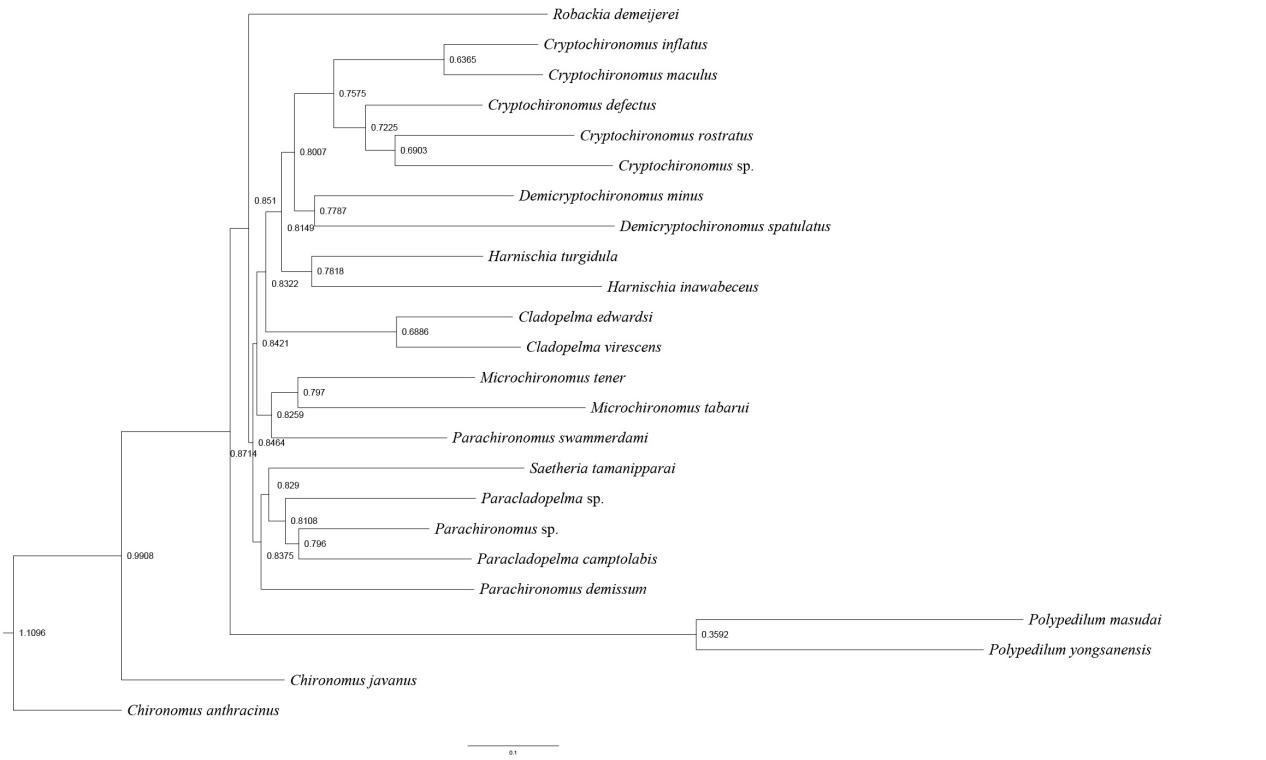


**Figure S5.** Phylogenetic tree of *Harnischia* generic complex, BI tree based on analysis cds_rrna in Partition.


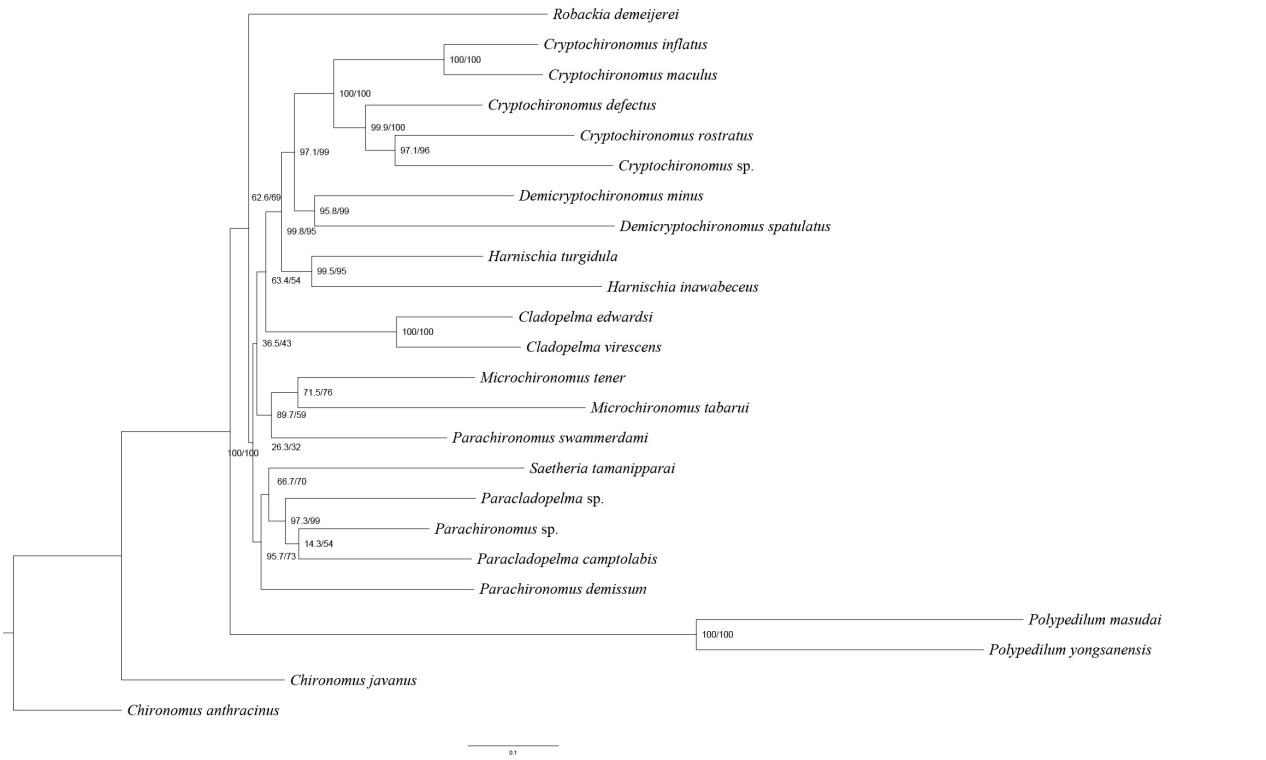


**Figure S6.** Phylogenetic tree of *Harnischia* generic complex, ML tree based on analysis cds_rrna in Partition.


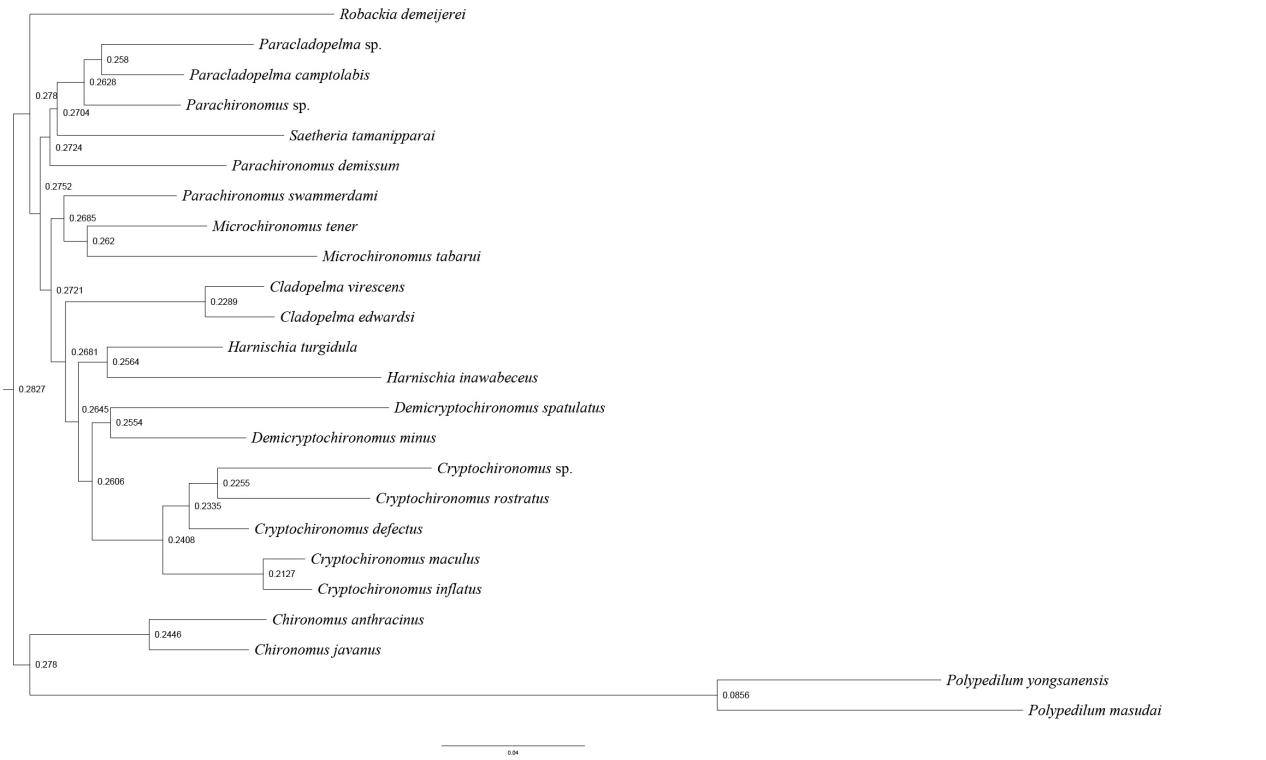


**Figure S7.** Phylogenetic tree of *Harnischia* generic complex, BI tree based on analysis cds12_fna in Partition.


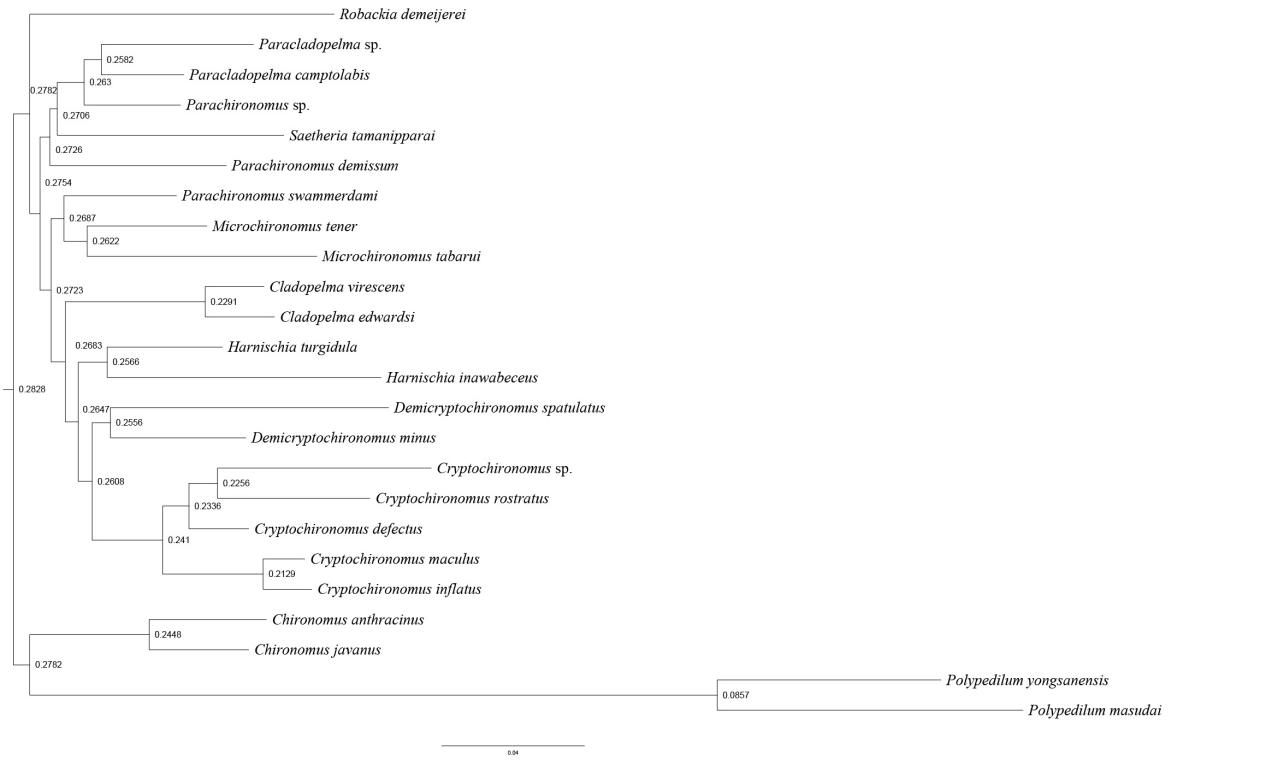


**Figure S8.** Phylogenetic tree of *Harnischia* generic complex, ML tree based on analysis cds12_fna in Partition.


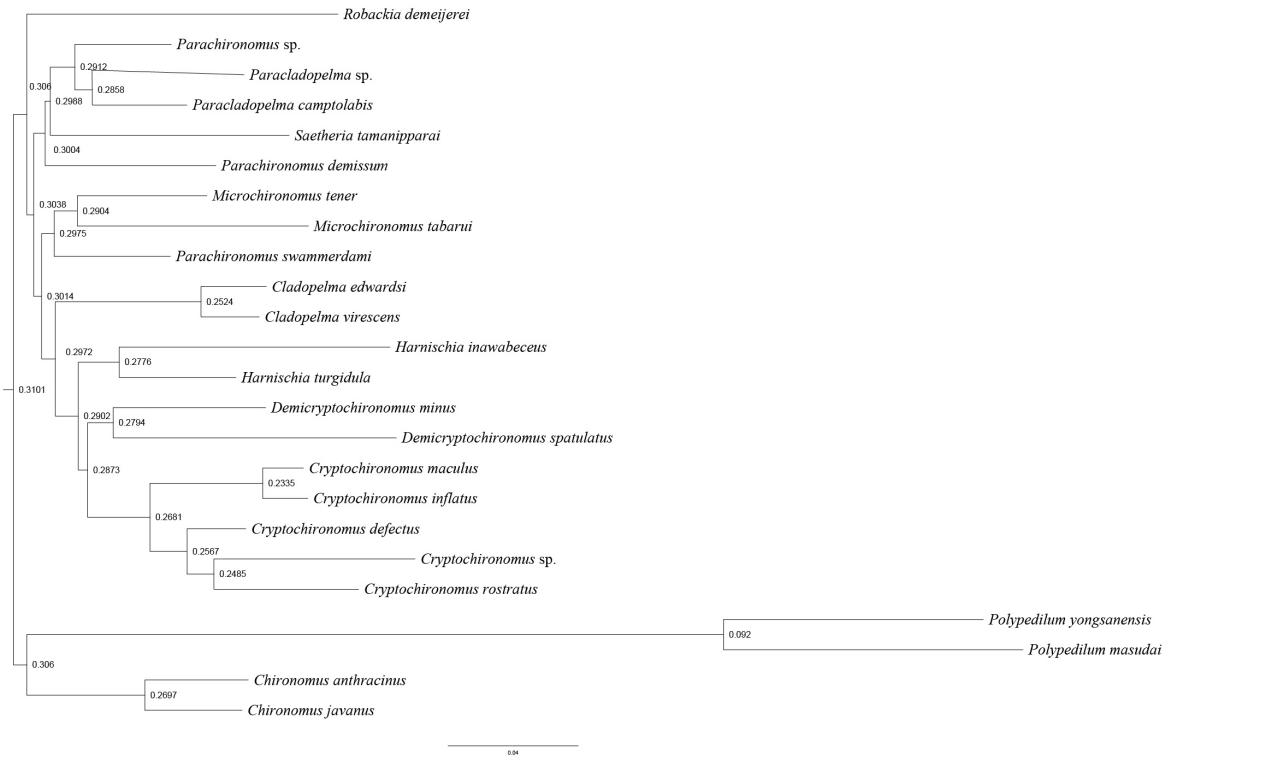


**Figure S9.** Phylogenetic tree of *Harnischia* generic complex, BI tree based on analysis cds12_rrna in Partition.


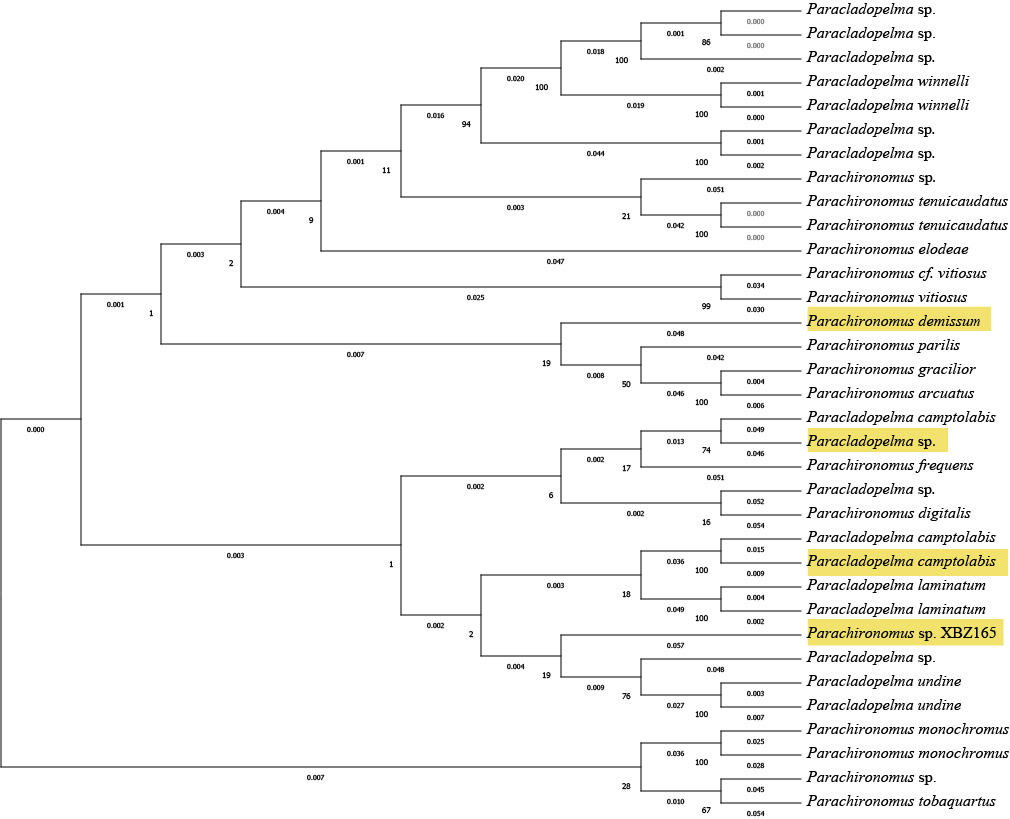


**Figure S10.** Phylogenetic trees of *Parachironomus* and *Paracladopelma* was constructed using the Neighbor-Joining method based on the *COI* gene sequences.


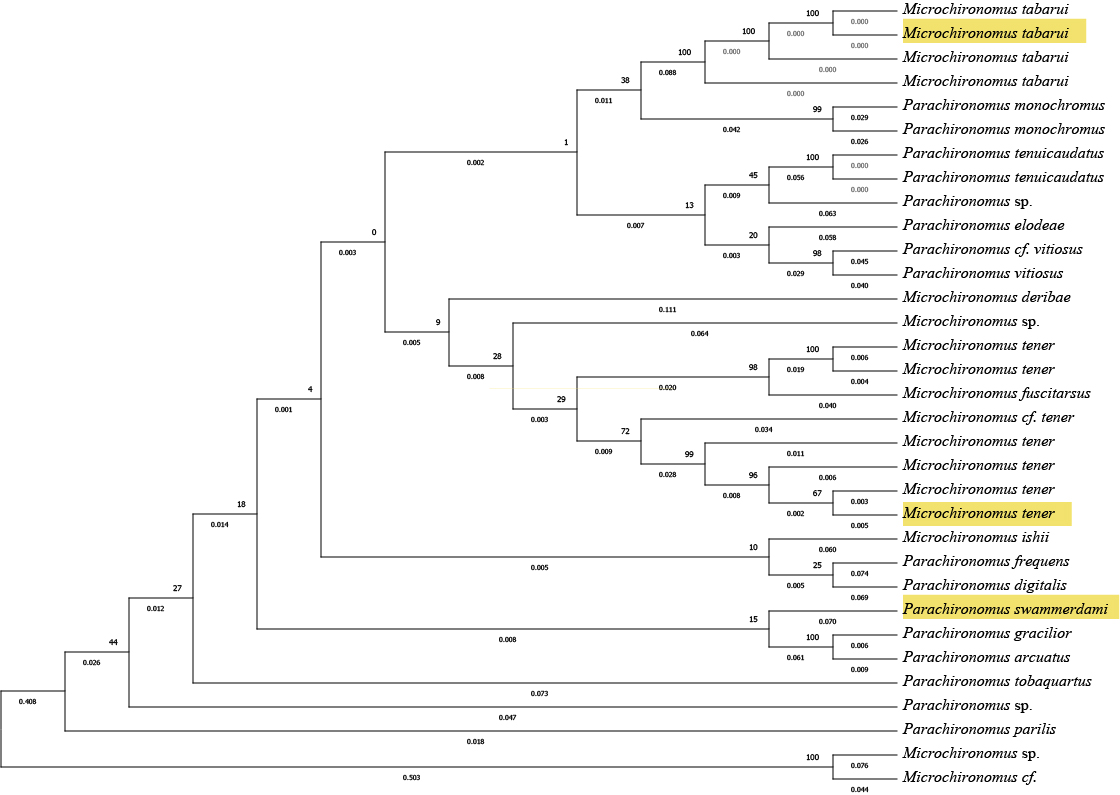


**Figure S11.** Phylogenetic trees of *Parachironomus* and *Microchironomus* was constructed using the Neighbor-Joining method based on the *COI* gene sequences.

**Table S1.** Final gene partitions for the Maximum Likelihood Phylogenetic analysis

| **Matrixs** | **Partition names** | **Best model** |
| --- | --- | --- |
| **cds_faa matrix** | *ATP6,COX1,COX2,COX3,CYTB*  *ATP8,ND2,ND3,ND6*  *ND1,ND4,ND4L,ND5* | mtART+R3  mtMet+F+R4  mtInv+R4 |
| **cds_fna matrix** | *ATP6,COX1,COX2,COX3,CYTB*  *ATP8,ND2,ND3,ND6*  *ND1,ND4,ND4L,ND5* | GTR+F+R4  TN+F+R4  GTR+F+R4 |
| **cds_rrna matrix** | *ATP6,COX1,COX2,COX3,CYTB,ND3*  *ATP8,ND2,ND6*  *ND1,ND4,ND4L,ND5*  *l-rrna,s-rrna* | GTR+F+R4  TIM3+F+R4  GTR+F+R4  GTR+F+R3 |
| **cds12_fna matrix** | *ATP6,COX1,COX2,COX3,CYTB*  *ATP8,ND6*  *ND1,ND2,ND3,ND4,ND4L,ND5* | TIM+F+I+G4  TPM3+F+I+G4  K3Pu+F+R3 |
| **cds12_rrna matrix** | *ATP6,COX1,COX2,COX3,CYTB*  *ATP8,ND6*  *ND1,ND2,ND3,ND4,ND4L,ND5*  *l-rrna,s-rrna* | TIM2+F+I+G4  TPM3+F+I+G4  K3Pu+F+R3  GTR+F+R3 |

**Table S2.** Mitogenomes of the 24 species used in this study.

| Subfamily | Species | GenBank Accession Number | Reference |
| --- | --- | --- | --- |
| Chironominae | *Cladopelma edwardsi* | PQ014460 | (Liu, et al. 2024) |
|  | *Cladopelma virescens* | PQ014464 | (Liu, et al. 2024) |
|  | *Cryptochironomus defectus* | PQ014461 | (Liu, et al. 2024) |
|  | *Cryptochironomus inflatus* | PP768808 | (Liu, et al. 2021) |
|  | *Cryptochironomus maculus* | PQ014454 | (Liu, et al. 2024) |
|  | *Cryptochironomus rostratus* | PQ014455 | (Liu, et al. 2024) |
|  | *Cryptochironomus* sp. | PQ014463 | (Liu, et al. 2024) |
|  | *Parachironomus swammerdami* | LC329152 | NCBI |
|  | *Demicryptochironomus minus* | PQ014456 | (Liu, et al. 2024) |
|  | *Demicryptochironomus spatulatus* | PQ014457 | (Liu, et al. 2024) |
|  | *Hanischia turgidula* | PQ014459 | (Liu, et al. 2024) |
|  | *Microchironomus tabarui* | MZ261913 | (Kong, et al. 2021) |
|  | *Microchironomus tener* | ON975027 | (Li, et al. 2022) |
|  | *Parachironomus demissum* | PQ867599 | (Liu, et al. 2025) |
|  | *Robackia demeijerei* | PQ867600 | (Liu, et al. 2025) |
|  | *Saetheria tamanipparai* | PQ867601 | (Liu, et al. 2025) |
|  | *Harnischia inawabeceus* | Pending | This study |
|  | *Parachironomus* sp. | Pending | This study |
|  | *Paracladopelma camptolabis* | Pending | This study |
|  | *Paracladopelma* sp. | Pending | This study |
|  | *Polypedilum masudai* | OK513041 | (Liu, et al. 2025) |
|  | *Chironomus javanus* | ON975025 | (Li, et al. 2022) |
|  | *Polypedilum yongsanensis* | OP950222 | (Liu, et al. 2025) |
|  | *Chironomus anthracinus* | ON975026 | (Li, et al. 2022) |

**Table S3.** Nucleotide composition of 4 mitogenomes

| Species | Whole Genome | | | | | PCG | | | | | tRNA | | | | |
| --- | --- | --- | --- | --- | --- | --- | --- | --- | --- | --- | --- | --- | --- | --- | --- |
|  | Length  (bp) | AT% | AT-  Skew | GC% | GC-  Skew | Length  (bp) | AT% | AT-  Skew | GC% | GC-  Skew | Length  (bp) | AT% | AT-  Skew | GC% | GC-  Skew |
| *Harnischia inawabeceus* | 15,941 | 78.23 | 0.02 | 21.76 | –0.22 | 11,220 | 76.66 | –0.18 | 23.34 | –0.07 | 15,940 | 78.24 | –0.19 | 21.76 | –1.34 |
| *Parachironomus* sp. | 15,789 | 78.89 | 0.02 | 21.11 | –0.18 | 11,220 | 78.04 | –0.18 | 21.96 | –0.03 | 1,489 | 79.72 | –0.17 | 20.28 | –0.56 |
| *Paracladopelma camptolabis* | 15,780 | 77.29 | 0.02 | 22.71 | –0.18 | 11,220 | 75.48 | –0.20 | 24.52 | –0.04 | 1,495 | 79.34 | –0.17 | 20.67 | –0.54 |
| *Paracladopelma* sp. | 15,850 | 78.22 | 0.03 | 21.78 | –0.19 | 11,223 | 76.74 | –0.19 | 23.26 | –0.03 | 1,493 | 79.7 | –0.13 | 20.3 | –0.60 |

| Species | rRNA | | | | | CR | | | | |
| --- | --- | --- | --- | --- | --- | --- | --- | --- | --- | --- |
|  | Length  (bp) | AT% | AT-  Skew | GC% | GC-  Skew | Length  (bp) | AT% | AT-  Skew | GC% | GC-  Skew |
| *Harnischia inawabeceus* | 2,211 | 84.44 | 0.00 | 15.56 | 0.41 | 697 | 95.40 | –0.10 | 4.30 | –0.53 |
| *Parachironomus* sp. | 2,186 | 84.24 | –0.01 | 15.77 | 0.37 | 656 | 94.97 | –0.12 | 5.03 | –0.33 |
| *Paracladopelma camptolabis* | 2,193 | 83.67 | 0.00 | 16.34 | 0.33 | 664 | 93.67 | –0.07 | 6.33 | –0.43 |
| *Paracladopelma* sp. | 2,191 | 84.45 | –0.01 | 15.55 | 0.37 | 627 | 96.01 | –0.01 | 3.98 | –0.12 |
