# Supplementary figures and images for: ﻿Phylogeny of the Harnischia generic complex (Diptera, Chironomidae) inferred from twenty whole mitogenomes
Source: Zookeys. 2026 Jan 19;1266:353–66. doi: 10.3897/zookeys.1266.162901 (PMC12835873; doi:10.3897/zookeys.1266.162901)

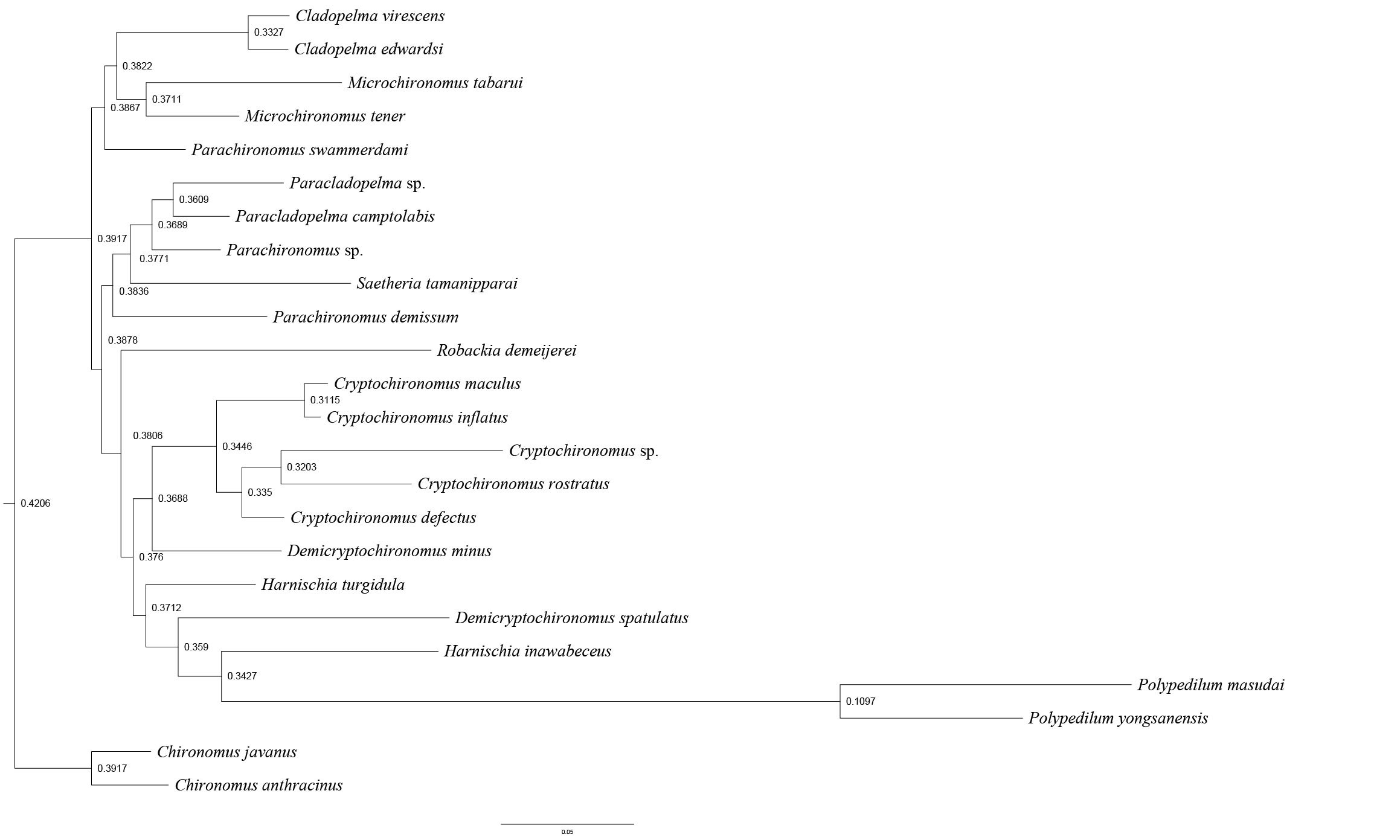

Supplement: Supplementary material 1 — Supplementary figures and tables [file zookeys-1266-353_article-162901__-s001.zip › Supplementary Materials/Figure S1.jpg]

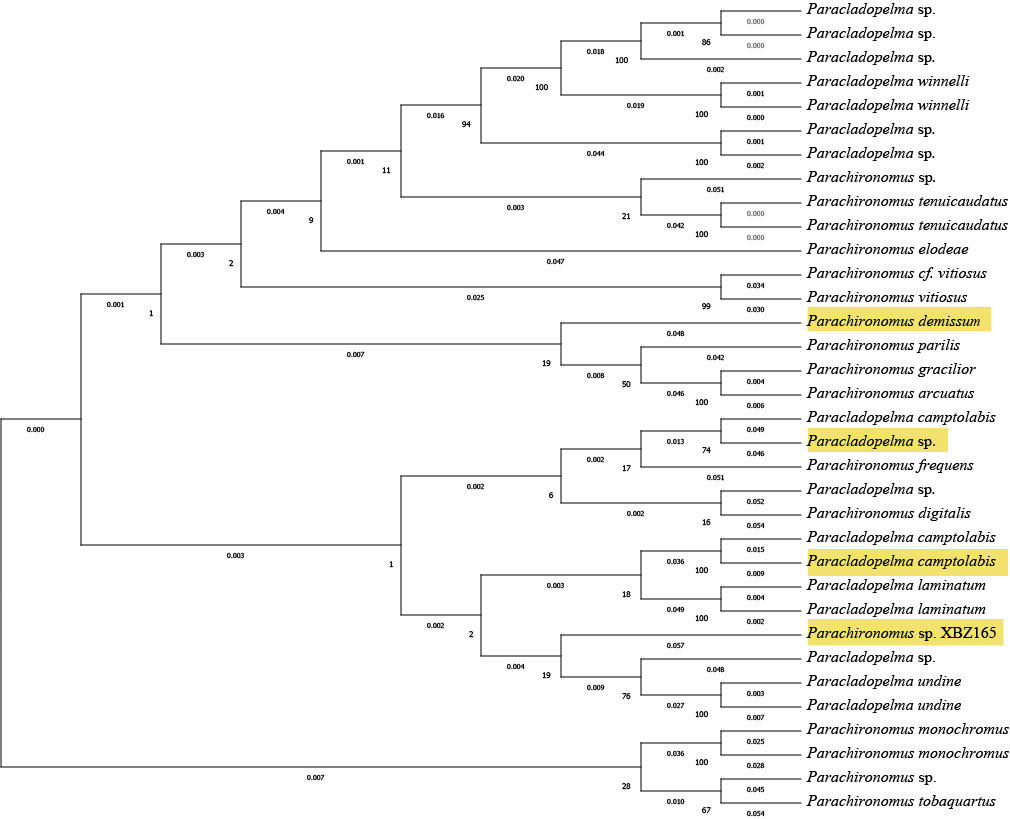

Supplement: Supplementary material 1 — Supplementary figures and tables [file zookeys-1266-353_article-162901__-s001.zip › Supplementary Materials/Figure S10.jpg]

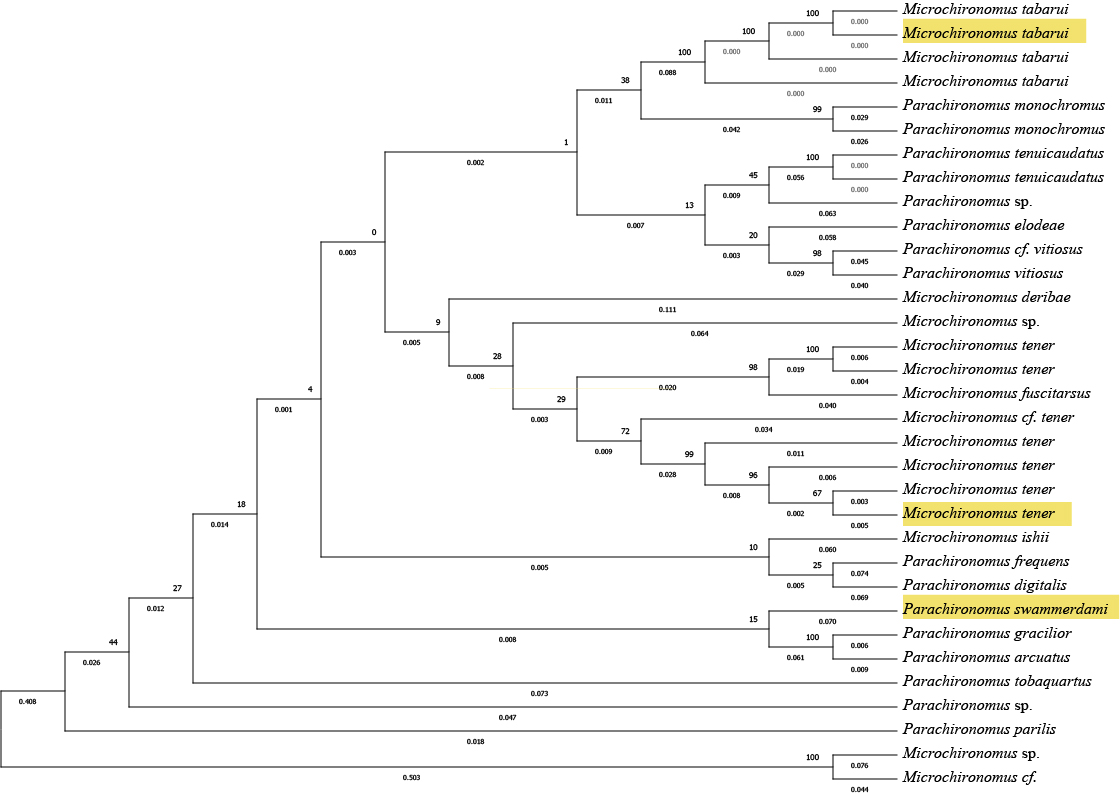

Supplement: Supplementary material 1 — Supplementary figures and tables [file zookeys-1266-353_article-162901__-s001.zip › Supplementary Materials/Figure S11.jpg]

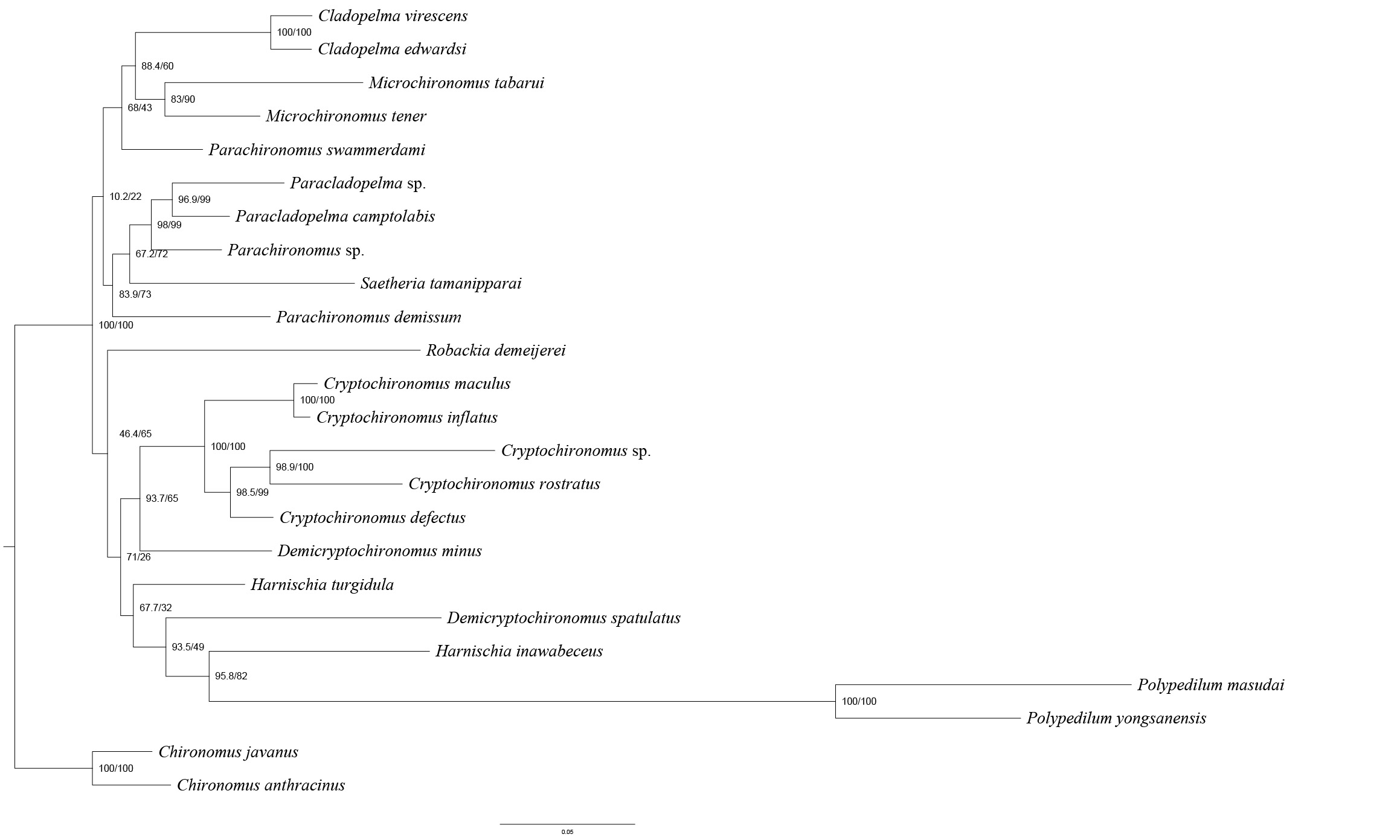

Supplement: Supplementary material 1 — Supplementary figures and tables [file zookeys-1266-353_article-162901__-s001.zip › Supplementary Materials/Figure S2.jpg]

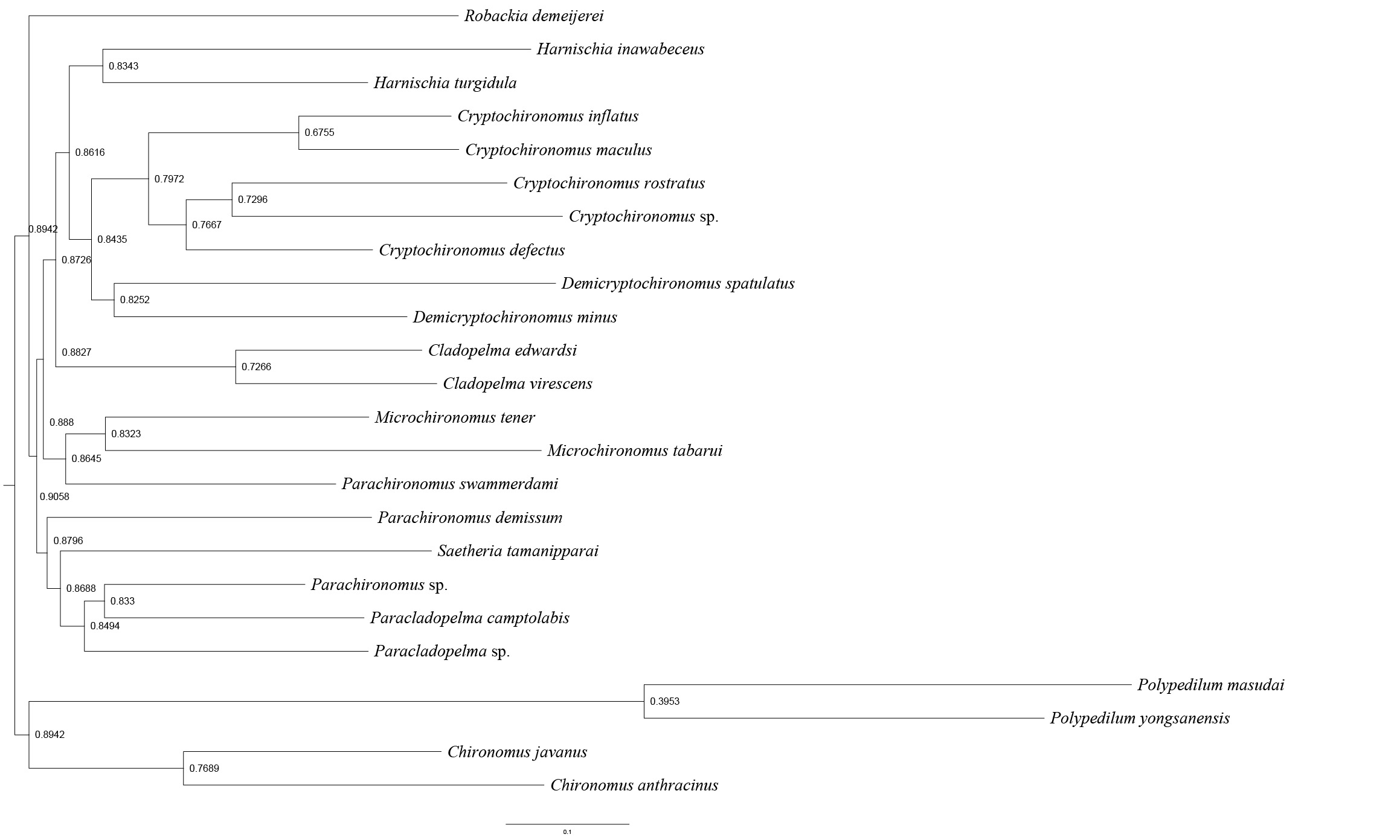

Supplement: Supplementary material 1 — Supplementary figures and tables [file zookeys-1266-353_article-162901__-s001.zip › Supplementary Materials/Figure S3.jpg]

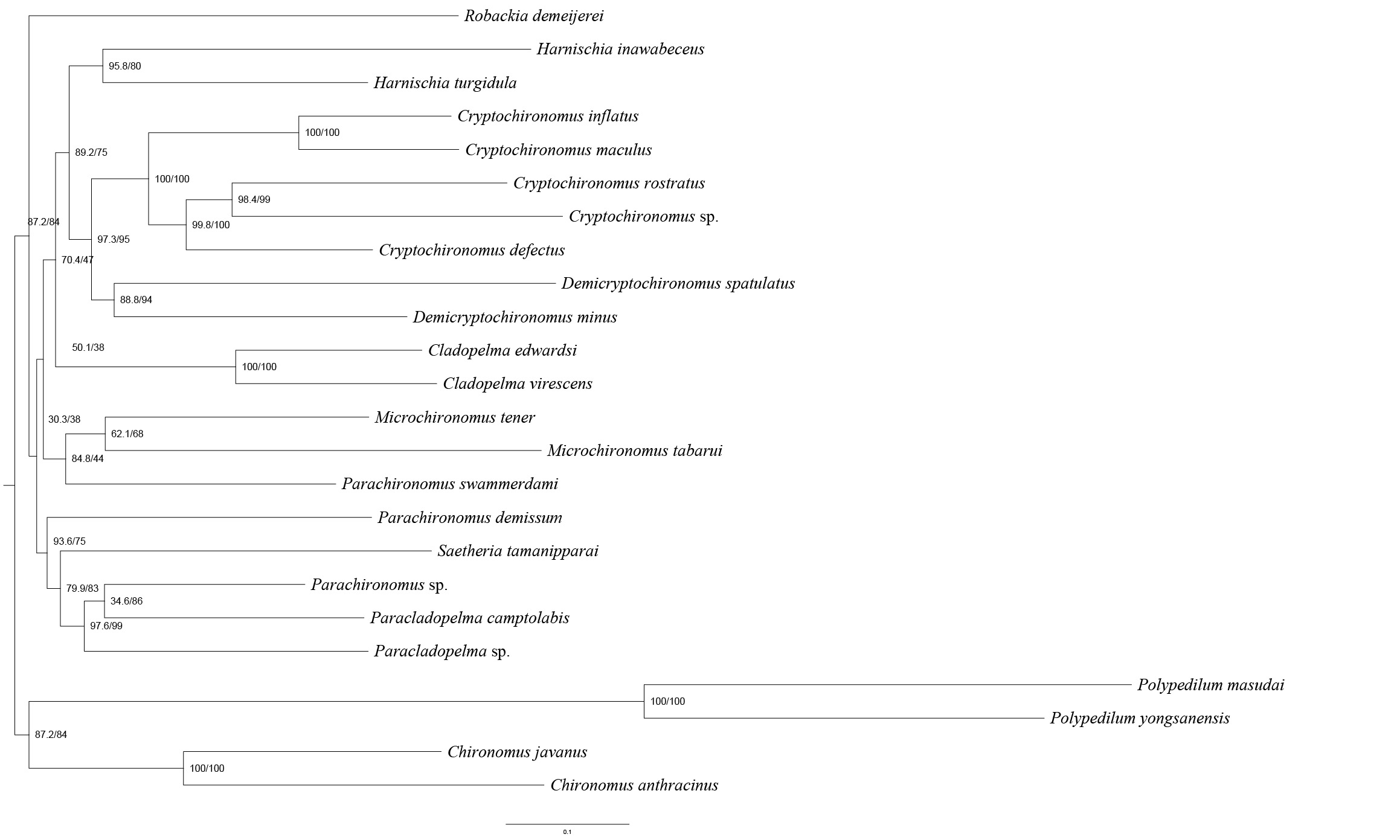

Supplement: Supplementary material 1 — Supplementary figures and tables [file zookeys-1266-353_article-162901__-s001.zip › Supplementary Materials/Figure S4.jpg]

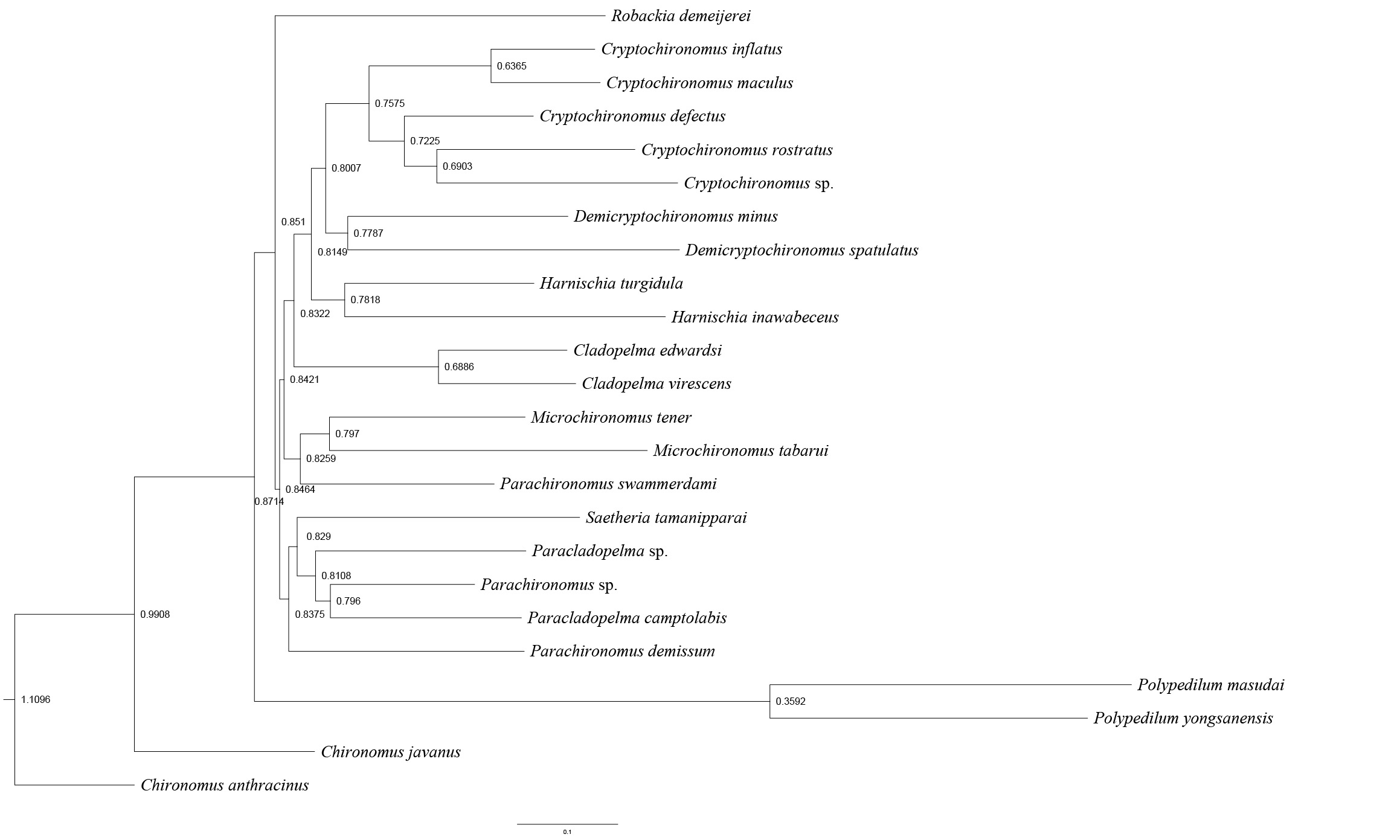

Supplement: Supplementary material 1 — Supplementary figures and tables [file zookeys-1266-353_article-162901__-s001.zip › Supplementary Materials/Figure S5.jpg]

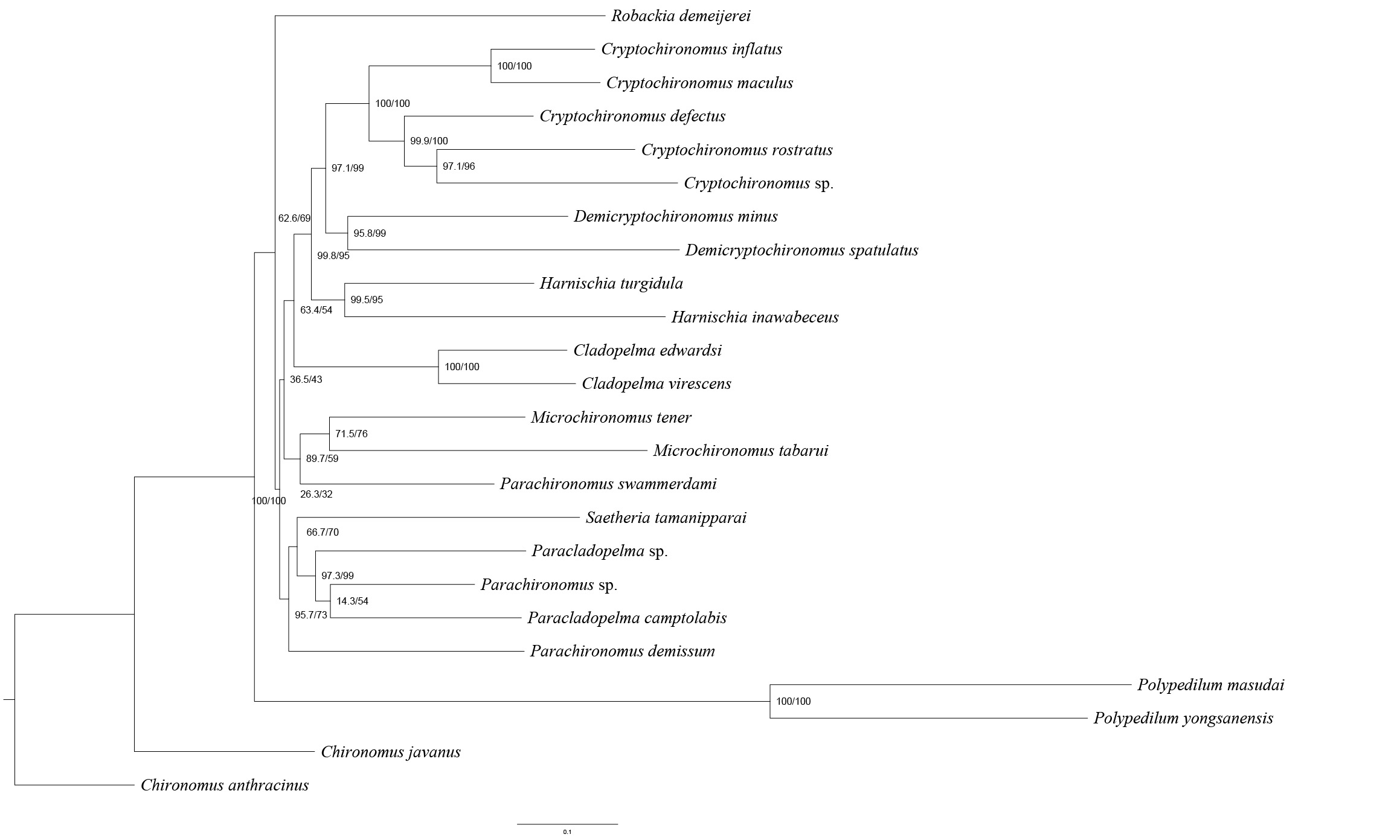

Supplement: Supplementary material 1 — Supplementary figures and tables [file zookeys-1266-353_article-162901__-s001.zip › Supplementary Materials/Figure S6.jpg]

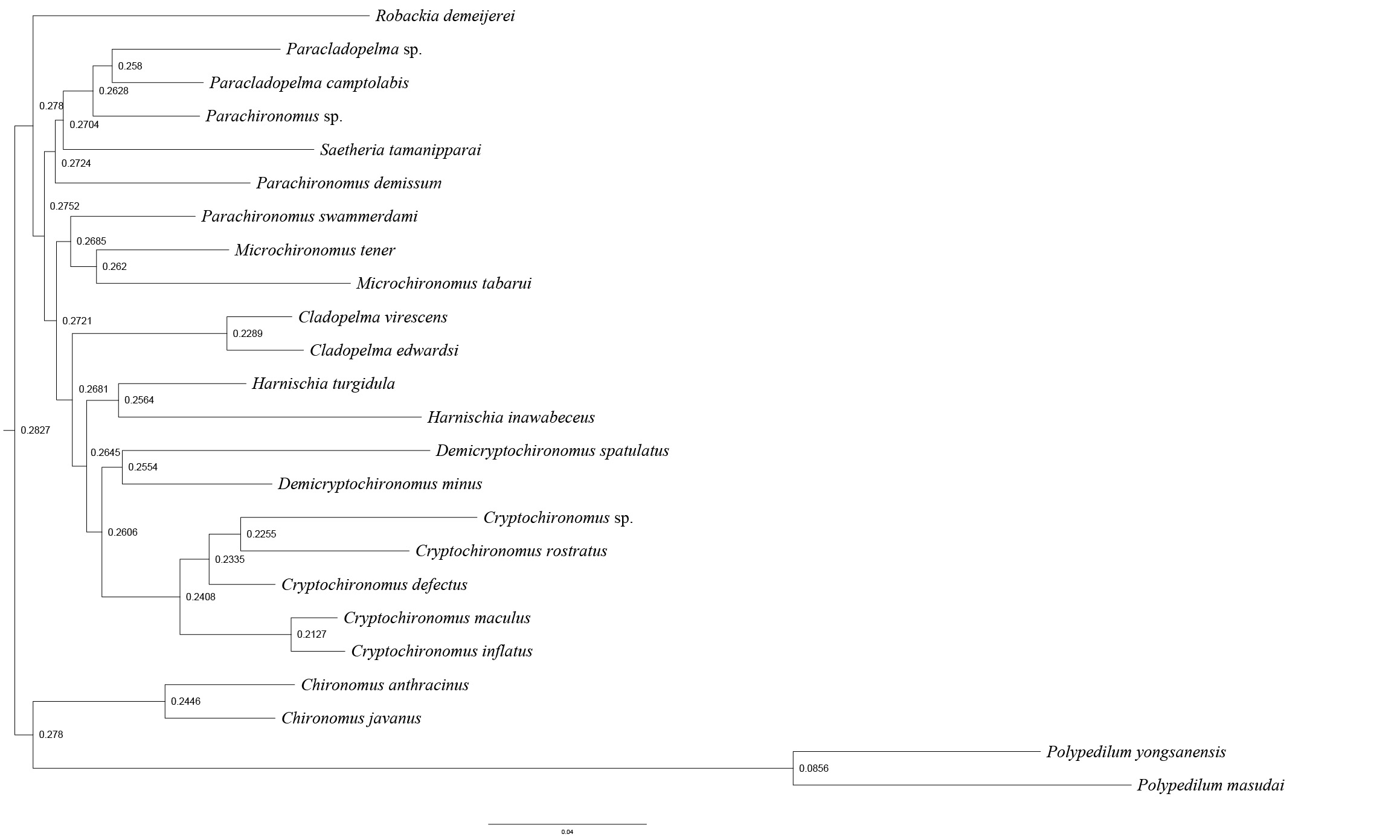

Supplement: Supplementary material 1 — Supplementary figures and tables [file zookeys-1266-353_article-162901__-s001.zip › Supplementary Materials/Figure S7.jpg]

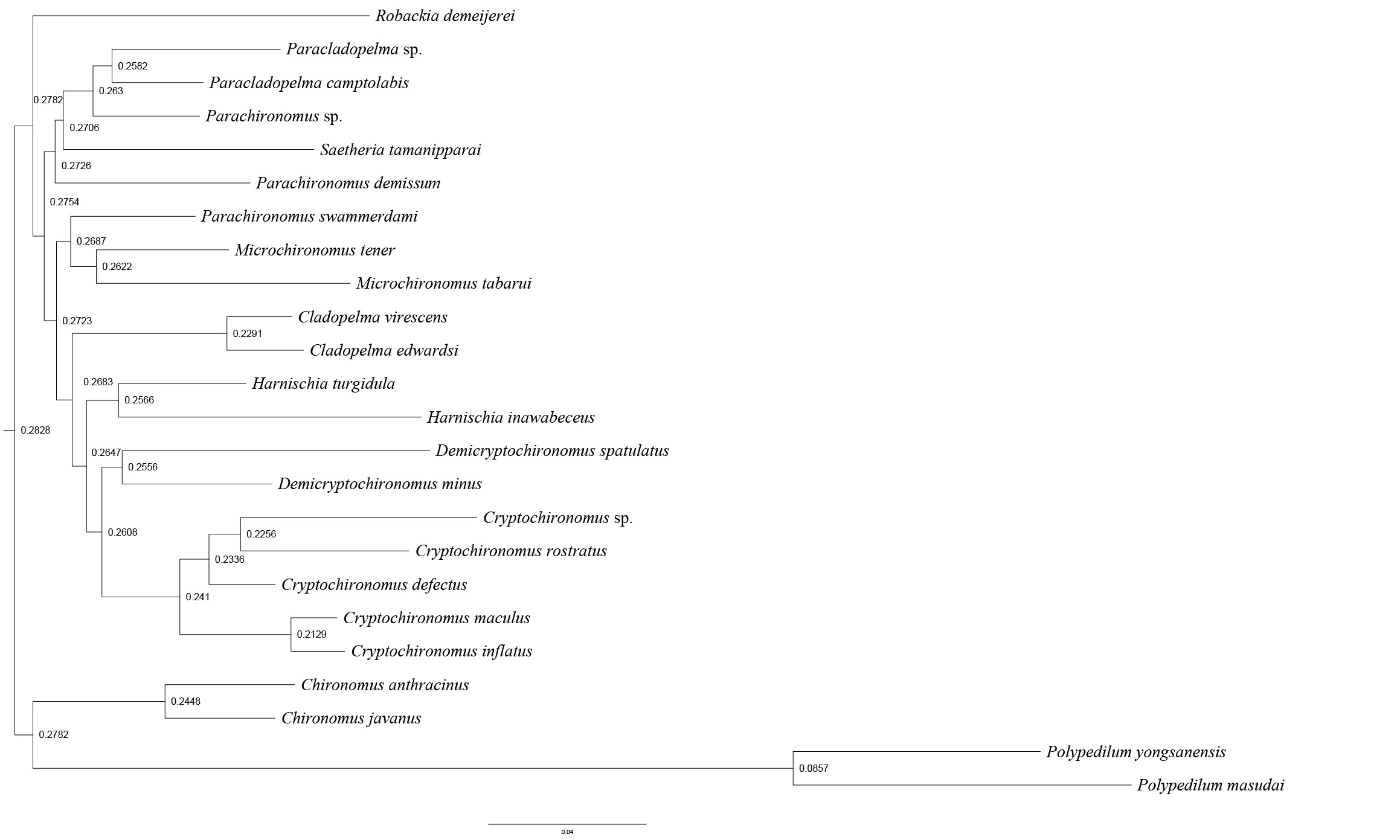

Supplement: Supplementary material 1 — Supplementary figures and tables [file zookeys-1266-353_article-162901__-s001.zip › Supplementary Materials/Figure S8.jpg]

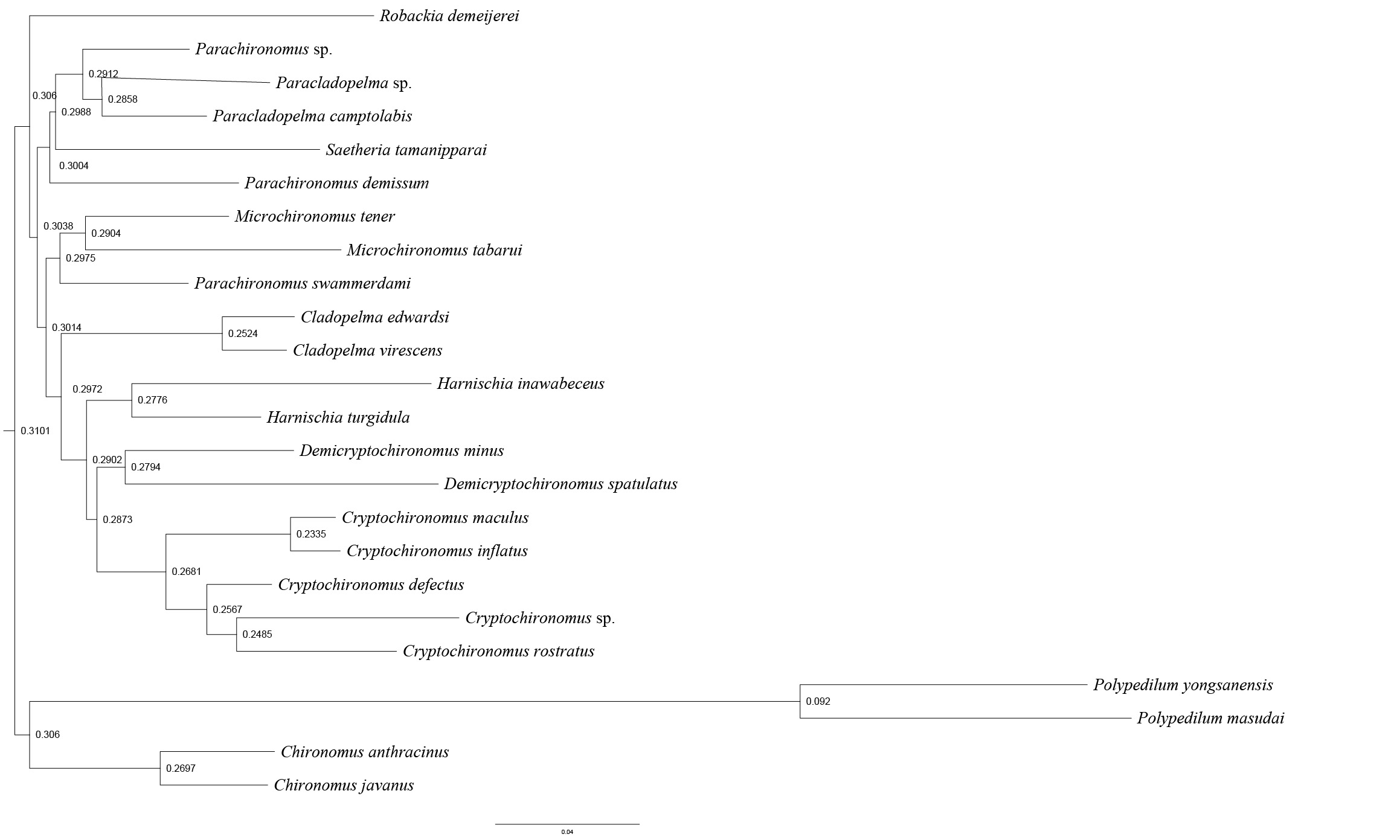

Supplement: Supplementary material 1 — Supplementary figures and tables [file zookeys-1266-353_article-162901__-s001.zip › Supplementary Materials/Figure S9.jpg]
